# *Phocaeicola dorei* YGMCC0564 attenuates HFD-induced glycolipid metabolic disorders in association with remodeling of the gut microbiota and bile acid profiles

**DOI:** 10.3389/fmicb.2026.1911556

**Published:** 2026-09-21

**Authors:** Zhili He, Ping Chen, Minlei Zhao, Wenfang Song, Shichang Zhang, Qiuxiang Lei, Xiaochi Jie, Jianguo Chen

**Affiliations:** 1 Beijing YuGen Pharmaceutical Co., Ltd., Beijing, China; 2 Dongguan Songshan Lake Donghua Hospital, Dongguan, China; 3 People’s Hospital of Xingtai, Xingtai, China

**Keywords:** bile acids metabolism, FXR-FGF15/19, GLMD, GPBAR1/TGR5, gut microbiota, *Phocaeicola dorei*

## Abstract

**Background:**

Glycolipid Metabolic Disorders (GLMD) are characterized by imbalances in glucose and lipid homeostasis, often presenting with severe clinical symptoms. Current pharmacotherapies such as orlistat and metformin show clear efficacy but are associated with long-term tolerability concerns. This study aimed to evaluate whether the next-generation probiotic candidate *Phocaeicola dorei* YGMCC0564 attenuates HFD-induced glycolipid metabolic disturbances in mice. We hypothesized that the protective effect of YGMCC0564 would be associated with modulation of bile acid metabolism and may be partly mediated by it.

**Methods:**

A high-fat diet (HFD)-induced mouse model of glycolipid metabolic disturbance was used, with YGMCC0564 supplementation initiated concurrently with HFD feeding in a prevention design. *Lactobacillus rhamnosus* GG and orlistat served as reference controls. Efficacy was assessed by body composition, histopathology, biochemical assays, and quantitative PCR, combined with 16S rRNA gene sequencing, non-targeted metabolomics, targeted quantification of fecal short-chain fatty acids, and cell-based functional assays. Safety was evaluated in acute and 4-week subchronic tests in immunodeficient and normal mice.

**Results:**

YGMCC0564 attenuated HFD-induced body weight gain, dyslipidemia, and hyperglycemia, reduced hepatic steatosis and gluconeogenic gene expression, and improved insulin resistance. Multi-omics analysis showed that YGMCC0564 reshaped the gut microbiota and restored fecal short-chain fatty acid concentrations that had been reduced by HFD feeding. The fecal bile acid profile was remodeled, with elevated lithocholic acid (LCA) and 3β,7α-dihydroxy-5-cholestenoate and reduced α-muricholic acid (α-MCA), chenodeoxycholic acid (CDCA), taurodeoxycholic acid (TDCA), and isoLCA. Intestinal expression of Fxr, Fgf15, Gpbar1/Tgr5, and Glp-1 was upregulated, consistent with enhanced bile acid receptor signaling. *In vitro*, a 1:1 mixture of LCA and 3β,7α-dihydroxy-5-cholestenoate, the two bile acids elevated by YGMCC0564 *in vivo*, activated FXR-FGF19 signaling in HT-29 intestinal cells and suppressed gluconeogenic gene expression and glucose output in HepG2 cells. Oral YGMCC0564 produced no obvious adverse effects in acute and subchronic tests in immunodeficient and normal mice.

**Conclusion:**

These findings indicate that YGMCC0564 may modulate FXR-FGF15/19 and GPBAR1/TGR5 signaling via the gut environment, thereby potentially contributing to its hypoglycemic and hypolipidemic effects.

## 1 Introduction

Glycolipid Metabolic Disorder (GLMD) is characterized by disrupted glucose and lipid metabolism. The core pathological mechanisms include neuroendocrine disorders, insulin resistance, oxidative stress, chronic inflammatory response, and a dysregulation of intestinal microbiome (Xiong et al., 2023). *Phocaeicola* is a genus of Gram-negative anaerobic bacteria within the *Bacteroidetes* phylum (Eom et al., 2026; García-López et al., 2019). This genus comprises multiple species, such as *Phocaeicola vulgatus* (*P. vulgatus*) and *Phocaeicola dorei* (*P. dorei*), which are common colonizing bacteria in the human intestine. Due to its unique complex heteropolysaccharide degradation capability and high bile salt hydrolase (BSH) enzyme activity, it primarily participates in physiological processes such as bile acid regulation, carbohydrate metabolism, short-chain fatty acid (SCFA) production, and lipid regulation (Le et al., 2022; Sun et al., 2023; Thomson et al., 2018).

Clinical studies have demonstrated a significant reduction in the abundance of *P. vulgatus* and *P. dorei* in patients with coronary artery disease (CAD). Furthermore, gavage with live *P. vulgatus* and *P. dorei* attenuated the formation of atherosclerotic lesions in atherosclerosis-prone mice (Yoshida et al., 2018). This evidence suggests that *P. vulgatus* and *P. dorei* are closely associated with human lipid metabolism. Additionally, another study revealed *P. dorei* BDX-01 alleviates dextran sulfate sodium (DSS)-induced experimental colitis (Sun et al., 2023). BDX-01 supplementation increased intestinal BSH activity and significantly enhanced intestinal bile acid excretion and deconjugation. Certain BAs act as farnesoid X receptor (FXR) agonists. Consistently, colonic FXR and fibroblast growth factor 15 (FGF15) were upregulated. Moreover, BDX-01 also downregulated the colonic proinflammatory cytokines pyrin domain-containing 3 (NLRP3), apoptosis-associated speck-like protein containing a CARD (ASC), cleaved caspase-1, and Interleukin-1 beta (IL-1β). Furthermore, in addition to BAs, bioactive compounds produced by *P. dorei* can directly activate FXR in intestinal epithelial cells (Zhang et al., 2015).

Upon activation, FXR triggers the secretion of the gut hormone FGF15/19 (human FGF19, mouse FGF15) from the ileum, which acts on the liver through the gut-liver axis to maintain the dynamic balance of glycolipid metabolism (Gadaleta and Moschetta, 2019; Kliewer and Mangelsdorf, 2015). Circulating through the portal vein to the liver, FGF15/19 activates the receptor complex of FGFR4 and β-klotho (KLβ) (Holt et al., 2003; Kurosu et al., 2007), mediating postprandial hepatic responses including the inhibition of bile acid and glucose synthesis and the stimulation of protein and glycogen synthesis (Inagaki et al., 2005; Kir et al., 2011). The metabolic effects of FGF15/19 are similar yet distinct from insulin, notably characterized by promoting protein and glycogen synthesis while inhibiting lipid synthesis.

Another major bile acid receptor, G protein-coupled bile acid receptor 1/Takeda G protein-coupled receptor 5 (GPBAR1/TGR5), is primarily expressed in enteroendocrine L cells (Shapiro et al., 2018; Sorrentino et al., 2020). Upon activation, GPBAR1/TGR5 stimulates glucagon-like peptide-1 (GLP-1) secretion, thereby improving insulin sensitivity and glucose homeostasis (Gao et al., 2026; Liu et al., 2024). Moreover, GPBAR1/TGR5 exerts anti-inflammatory effects, contributing to the maintenance of glucose and immune homeostasis (Liu et al., 2024; Sun et al., 2025; Yan et al., 2025).

Despite advances in understanding GLMD pathogenesis, its clinical management remains challenging. Patients with GLMD often present with severe metabolic symptoms arising from imbalances in glucose and lipid homeostasis. Although conventional pharmacotherapies such as orlistat and metformin have demonstrated clear efficacy, their long-term adverse effects cannot be overlooked, highlighting the urgent need to develop safe and effective natural probiotic therapeutics. In this context, *P. dorei* has emerged as a promising probiotic candidate; however, while its beneficial effects have been documented in colitis (Sun et al., 2023; Zhang et al., 2015) and atherosclerosis (Yoshida et al., 2018), its therapeutic potential in GLMD has not been systematically investigated. Furthermore, although the FXR–FGF15/19 and GPBAR1/TGR5 pathways are established mediators of bile acid signaling in metabolic regulation, the integrated mechanisms by which *P. dorei* remodels the gut microbiota, alters the bile acid pool, and activates downstream receptors to ameliorate GLMD remain elusive, particularly from a multi-omics perspective.

Our previous research demonstrated that the strain *P. dorei* YGMCC0564 exhibits high BSH activity and cholesterol conversion rates, with potential lipid-lowering effects (He et al., 2023). Building upon these findings, the present study aims to: (1) evaluate the effects of *P. dorei* YGMCC0564 on attenuating HFD-induced glycolipid metabolic disturbances in mouse models; (2) characterize the alterations in gut microbiota and bile acid profiles induced by YGMCC0564 using multi-omics approaches; and (3) explore the potential mechanisms by which YGMCC0564 may modulate the GPBAR1/TGR5 and FXR–FGF15 signaling pathways. Based on multi-omics association analysis and cellular validation, we hypothesize that YGMCC0564 attenuates HFD-induced glycolipid metabolic disorders by reshaping the gut microbiota and modulating bile acid metabolism, thereby concurrently activating the GPBAR1/TGR5 and FXR–FGF15 signaling pathways.

## 2 Materials and methods

### 2.1 Analysis of GMrepo database

The relative microbial abundance of Phocaeicola species associated with the obese phenotype was obtained from the GMrepo database. The relative abundance of 20 main species was calculated for each sample, with the total abundance value being 100%. Relative abundance of *Phocaeicola* species from fecal samples of healthy and obese patients was obtained using the GMRepo RESTful APIs for R^1^ and RStudio^2^ software. The data source information was traced, and the sample descriptions were identified to assess the data quality. In the normal population, 1,000 samples with species abundance greater than 0 were randomly selected as the control group, while in the obese population, all samples with species abundance greater than 0 were selected as the experimental group. To avoid false positives caused by confounding factors, we selected datasets with large sample sizes for statistical analysis. Although confounding factors such as age, gender, and dietary habits are difficult to control in this database comparison, the outcome variable of interest is the difference in gut microbiota composition. The taxonomic nomenclature of the microbiome adopts the updated classification names from the GMrepo database. Subsequently, the relative abundance of Phocaeicola species in healthy and obese patients was extracted, and the differences in abundance among different populations were analyzed.

### 2.2 Bacterial strains and culture conditions

*Phocaeicola dorei* YGMCC0564 (also known as *Phocaeicola dorei* YG·UP188) was provided by the Culture Collection of Microbiology, YuGen Pharmaceutical Co., Ltd. (Beijing, China). *Lactobacillus rhamnosus* GG (LGG) was provided by ATCC 53103. Strains were removed from the −80 °C refrigerator, quickly thawed and transferred to fresh Brain Heart Infusion with Supplement (BHIS) medium, and cultured in a 37 °C anaerobic environment. After successful resuscitation, the strains were cultured on BHIS plate medium, and the single clone was transferred to fresh BHIS medium again. When OD_600_ = 1.0, the strains were passaged 2–3 times to obtain the strain with sufficient vitality. Strains were grown in expanded volumes and then centrifuged for 10–15 min at 7,500 rpm. After discarding the supernatant, the pellet was washed once with pre-cooled PBS. Finally, the pre-cooled 20% glycerol solution was added for resuspension, and the frozen stock solution was prepared and put into the −80 °C refrigerator. The bacterial solution was diluted with normal saline before gavage and adjusted to the number of live bacteria (1 × 10^10^ CFU/mL).

### 2.3 HFD-animal experiments

Specific Pathogen-Free (SPF) C57BL/6J male mice (8 weeks of age) were purchased from Beijing Vital River Laboratory Animal Technology Co., Ltd. and were housed at temperature 23 ± 3 °C with 50% ± 10% humidity (12 h light/12 h dark cycle). All mice were allowed to eat and drink ad libitum, and molar rods were placed in the housing cages. Mice were group-housed with 5 animals per cage (a total of 10 cages for the 50 experimental mice) under the stated SPF conditions. Mice were randomly divided into 5 groups: normal diet group (ND), high fat diet group (HFD), high fat diet add positive control group (Orlistat), high fat diet add LGG group (LGG), and high fat diet add YGMCC0564 group (YGMCC0564), 10 mice per group. Group assignment was performed by simple randomization at the individual-mouse level. Cages were not systematically allocated to a specific treatment group, and cage was not included as a covariate or blocking factor in the statistical analysis. LGG was included as a representative first-generation (classical) probiotic comparator. As one of the most extensively characterized probiotic strains with an established safety record, LGG serves as a benchmark for conventional probiotic intervention. In the present study, YGMCC0564 was therefore evaluated against two distinct references, namely the pharmacological positive control (Orlistat) and the first-generation probiotic LGG, so that its effects could be comparatively assessed against both conventional probiotic and pharmacological interventions. After the 1-week adaptation period, except for the ND group, the other groups were fed with a high-fat diet in which fat provided 48.5% of total calories (kcal%) (Beijing Botai Hongda, feed ingredients shown in Supplementary Table 1) for 8 weeks. The Orlistat group received 1.2 mg of Orlistat per day, LGG mice were gavaged 1 × 10^9^ CFU LGG daily, and 1 × 10^9^ CFU YGMCC0564 were gavaged daily in the YGMCC0564 group per mouse. An equivalent volume of PBS was used as a control in the ND and HFD groups. The above mice were administered continuously for 8 weeks and weighed once a week. At week 8, mouse feces were collected, stored at −80 °C and followed by fecal microbial diversity detection. At the 8th week of modeling, the mice fasted overnight. Next day, mice were anesthetized with isoflurane and sacrificed by exsanguination. Blood samples were tested for fasting glucose, while serum was collected and stored at −80 °C for future serological testing. The mouse was immobilized, the shoulder blades were exposed, and the interscapular brown fat tissue (BAT) was taken. Abdominal cavity and chest cavity were then opened, liver, colon, cecum contents, inguinal white fat tissue (iWAT) and epididymal white fat tissue (eWAT) were removed, weighted and frozen immediately in liquid nitrogen, and stored at −80 °C until processing. Small pieces of liver, iWAT and BAT were fixed with 4% paraformaldehyde, respectively, and stored at room temperature.

To determine the intervention dose for the main experiments, a preliminary dose-range experiment was conducted before the main study. Male C57BL/6J mice (8 weeks of age, *n* = 8 per group) were fed the high-fat diet described above and concurrently received daily oral gavage of YGMCC0564 at 10^8^, 10^9^, or 10^10^ CFU/day for 8 weeks. A high-fat diet control group was included in parallel. Body weight was recorded weekly throughout the experimental period, and the data are presented in Supplementary Figure 4.

### 2.4 Toxicity to normal mice and nude mice

Referring to the study by Hou et al. (2023), we conducted strain toxicity experiments. For the acute toxicity assessment, 40 BALB/c nude mice (20 females and 20 males, 6–8 weeks old) were purchased from Beijing Vital River Laboratory Animal Technology Co., Ltd. and randomly assigned to a control (Con) group and a YGMCC0564 group (*n* = 20 per group, with 10 males and 10 females each). Mice in the YGMCC0564 group received a daily oral gavage of 1 × 10^9^ CFU of YGMCC0564 suspended in 0.1 mL normal saline for 3 consecutive days, while the Con group was administered an equivalent volume of normal saline. General condition was monitored, and body weight was recorded daily for 7 days. At the end of the test, the mice were fasted for 24 h, anesthetized with isoflurane, and blood samples were collected for routine hematological and biochemical analyses. Subsequently, the animals were euthanized, and major organs were harvested for histopathological examination.

For the chronic toxicity evaluation, 60 BALB/c mice (30 females and 30 males, 6–8 weeks old) were obtained from the same supplier and randomly divided into three groups (*n* = 20 per group, 10 males and 10 females each): a Con group, a low-dose YGMCC0564 group (YGMCC0564-L), and a high-dose YGMCC0564 group (YGMCC0564-H). Mice in the YGMCC0564-L and YGMCC0564-H groups were intragastrically given 1 × 10^9^ CFU and 1 × 10^10^ CFU of YGMCC0564 in 0.1 mL normal saline daily, respectively, for 1 month, whereas the Con group received the same volume of normal saline. General health status was observed daily, and body weight was measured twice per week. At the end of the experiment, after 24 h of fasting, the mice were anesthetized with isoflurane, blood was drawn for routine hematological and biochemical assays, and the animals were then euthanized to collect organs for histopathological analysis. The complete grouping, dose, and control design of both safety tests are summarized in Supplementary Table 7.

### 2.5 Pathological detection

Liver, iWAT, BAT and colon samples were fixed in 4% paraformaldehyde, dehydrated in ethanol, embedded in paraffin, sectioned at 5 μm and stained with hematoxylin and eosin (H&E). The degree of steatosis was assessed based on the level of involvement in liver parenchyma using the following criteria: 0 (<5%), 1 (5–33%), 2 (33–66%), or 3 (>66%). Inflammation was evaluated by considering the overall presence of inflammatory foci using the following criteria: 0 (no foci), 1 (<2 foci per 200 X field), 2 (2–4 foci per 200 X field), or 3 (>4 foci per 200 X field). The cell diameter in iWAT and BAT was measured using specialized software. The software randomly selected 10 fields of view for analysis.

### 2.6 Blood glucose and serum insulin assay

A drop of blood was taken from the mice and put on the test paper, and the fasting blood glucose value was measured with a glucometer. Insulin levels were detected according to the instructions of the Serum Insulin Assay Kit (Biyun Tian, China). Insulin resistance index (HOMA-IR) = fasting insulin (μU/mL) × fasting blood glucose (mmol/L)/22.5.

### 2.7 Biochemical assays

Precooled absolute ethanol lysate was added at a mass/volume ratio of 1:9 to 100 mg of liver tissue for homogenization. Total cholesterol (TC) and triglyceride (TG) in serum and liver homogenate were measured using a fully automated biochemical analyzer (Myriad, China).

### 2.8 Gene expression quantification by quantitative PCR

Total RNA was isolated from liver, iWAT, and BAT using a total RNA purification kit (TransGen Biotech, China). Liver tissue was homogenized in lysis buffer using a tissue homogenizer, then centrifuged at 12,000 × g for 5 min at 4 °C, and the supernatant was used for RNA purification per the kit protocol. For fat samples, Trizol lysis was followed by centrifugation at 12,000 rpm for 10 min at 4 °C to increase the purity of the extracted RNA. Reverse Transcription Kit (Takara, Beijing, China) was used for reverse transcription to obtain cDNA samples. qPCR detection was performed using the Rapid Fluorescence PCR Kit (Tiangen, China) and Fluorescence Quantitative PCR Amplifier (Roche® 480, Switzerland). Gene expression was normalized to β-actin, which served as the reference gene. The primer sequences for each target gene are listed in Supplementary Table 2. The amplification efficiency of every primer pair was determined from standard curves generated by 10-fold serial dilutions of pooled cDNA, and all primer pairs showed efficiencies between 90 and 120%. For each group, 10 mice were included as biological replicates, and each sample was assayed in triplicate (technical replicates). Relative mRNA expression was calculated by the 2^−ΔΔCt^ method with β-actin as the normalizer.

To indirectly reflect the intestinal abundance changes of YGMCC0564 and LGG, we quantified the fecal abundance of their corresponding species, *P. dorei* and *L. rhamnosus*, using genomic DNA-based species-specific primers (BD_0416 (He et al., 2023) and LGG-1, the BD_0416 and LGG-1 primer sequences are provided in Supplementary Table 2). Fecal samples were collected at 0 and 8 weeks, and genomic DNA was extracted. The gene copy numbers of *P. dorei* and *L. rhamnosus* in the samples were detected by qPCR. For absolute quantification, plasmids containing the BD_0416 and LGG-1 gene fragments were gradient-diluted to known concentrations, and standard curves were plotted using Ct values to calculate the absolute abundance of the target strains in each sample.

### 2.9 16S rRNA analysis of the gut microbiota

Fresh mouse fecal samples were collected in sterile EP tubes, flash-frozen in liquid nitrogen, and stored at −80 °C. Total genomic DNA was extracted using the OMEGA Soil DNA Kit. PCR amplification was carried out with universal primers targeting the V3-V4 hypervariable regions of the bacterial 16S rRNA gene. A no-template control (CK) was included in every PCR run and verified by agarose gel electrophoresis (Supplementary Figure 1). Paired-end sequencing was performed on the Illumina MiSeq PE300 platform by Majorbio Bio-Pharm Technology Co. Ltd. (Shanghai, China). Raw sequencing reads were quality-filtered using fastp (v1.2.11) and merged using FLASH (v1.2.11). After quality filtering, an average of 45,344 sequences per sample (range 31,045–62,861) were retained for downstream analysis. Rarefaction curves of the observed operational taxonomic units reached an asymptote, indicating sufficient sequencing depth (Supplementary Figure 2F), and all samples were rarefied to 30,000 sequences per sample before the alpha and beta diversity analyses. Quality-filtered sequences were clustered into operational taxonomic units at 97% similarity using UPARSE, with chimeric sequences removed. Taxonomic annotation of operational taxonomic unit (OTU) representative sequences was performed using the RDP Classifier (version 2.13^3^) against the Silva 16S rRNA database (v138) with a confidence threshold of 70%. Alpha diversity indices, including the Chao index (estimating richness) and the Shannon index (estimating diversity), were calculated using Mothur (v1.30.2). Beta diversity was evaluated by principal coordinate analysis based on Bray–Curtis dissimilarity. Group differences in beta diversity were assessed by permutational multivariate analysis of variance (PERMANOVA), which indicated significant differences among the five groups (*R*^2^ = 0.209, *p* = 0.001, Supplementary Figure 2D), and the homogeneity of multivariate dispersions was verified by permutational dispersion analysis (PERMDISP), which confirmed homogeneous dispersions across groups (*p* ≥ 0.05). To identify bacterial taxa with significantly different abundances between groups from phylum to genus level, Linear discriminant analysis Effect Size analysis was performed, using a linear discriminant analysis score threshold of >3.0. Spearman’s rank correlation analysis was conducted to explore associations between key microbial genera and physiological indices.

### 2.10 Metabolomics analysis

Metabolomics analysis in this study followed previous research protocols (He et al., 2024). Metabolite analysis utilized LC–MS/MS with a Thermo UHPLC-Q Exactive HF-X system equipped with an ACQUITY HSS T3 column (100 mm × 2.1 mm i.d., 1.8 μm; Waters, United States) at Majorbio Bio-Pharm Technology Co. Ltd. (Shanghai, China). Mass spectrometry data collection entailed both positive and negative ion scanning across a mass range of 70–1,050 m/z. Subsequent stages comprised metabolite targeting, automated MS/MS mode for fragment ion analysis, and differentiation of metabolites in two cohorts, followed by pathway identification through metabolic enrichment and Kyoto Encyclopedia of Genes and Genomes (KEGG) database analysis. Fecal samples were extracted with prechilled methanol/acetonitrile (1:1, v/v) containing 0.02 mg/mL L-2-chlorophenylalanine as an internal standard. The homogenized mixtures were sonicated, centrifuged, and filtered through 0.22 μm membranes prior to injection. Pooled quality control (QC) samples were prepared by mixing equal volumes of each sample extract; five QC injections were performed at the beginning of the analytical sequence to equilibrate the system, followed by one QC injection every six biological samples throughout the run and a final QC injection at the end, thereby enabling continuous monitoring of signal drift. Biological samples were analyzed in randomized order with QC samples evenly interspersed. Signal normalization was achieved by combining internal standard normalization with total ion current (TIC) normalization, and QC-based locally estimated scatterplot smoothing (QC-RLSC) regression was subsequently applied to correct for injection order–dependent signal drift. Features exhibiting a relative standard deviation (RSD) > 30% in the QC samples were excluded, and missing values were imputed with half of the minimum positive value across all samples. Differential metabolites were identified using the following criteria: variable importance in projection (VIP) > 1.0, fold change ≥ 1.5 or ≤ 0.667, and *p* < 0.05 (Student’s *t*-test). Metabolite annotation was performed in accordance with the Metabolomics Standards Initiative (MSI) guidelines; lithocholic acid (LCA) and 3β,7α-dihydroxy-5-cholestenoate were annotated based on high-resolution accurate mass (mass error < 10 ppm), isotopic pattern matching, and MS/MS fragmentation comparison against the HMDB and METLIN databases, corresponding to MSI Level 2 (putative annotation).

### 2.11 Targeted quantification of SCFAs by GC–MS

Fecal SCFA concentrations were determined by targeted gas chromatography–mass spectrometry (GC–MS) at Majorbio Bio-Pharm Technology Co. Ltd. (Shanghai, China). Briefly, 25 mg of each fecal sample was homogenized in 500 μL of water containing 0.5% phosphoric acid, ground at 50 Hz for 3 min twice, sonicated for 10 min, and centrifuged at 4 °C and 13,000 × g for 15 min. A 200 μL aliquot of the supernatant was extracted with 200 μL of n-butanol containing 2-ethylbutyric acid (10 μg/mL) as the internal standard, and the mixture was vortexed for 10 s, sonicated at low temperature for 10 min, and centrifuged at 4 °C and 13,000 × g for 5 min. The supernatant was then transferred to injection vials for analysis.

GC–MS analysis was performed on an Agilent 8890B gas chromatograph coupled to an Agilent 5977B mass selective detector equipped with an electron impact ionization source (70 eV). Analytes were separated on an HP-FFAP capillary column (30 m × 0.25 mm × 0.25 μm) with 99.999% helium as the carrier gas at a constant flow of 1 mL/min. The oven temperature was programmed to hold at 80 °C, rise to 120 °C at 40 °C/min, then rise to 200 °C at 5 °C/min, and finally hold at 220 °C for 3 min. The injection volume was 1 μL in split mode (10:1) with an inlet temperature of 180 °C, and the ion source and quadrupole were maintained at 230 °C and 150 °C, respectively. Data were acquired in selected ion monitoring (SIM) mode.

Quantification was performed against seven-point calibration curves prepared from authentic standards of eight SCFAs (acetic, propionic, butyric, isobutyric, valeric, isovaleric, hexanoic, and isohexanoic acid), with 2-ethylbutyric acid as the internal standard. Peak identification and integration were performed using MassHunter quantitative software (Agilent, United States, v10.0.707.0), and all integrations were checked manually. SCFA concentrations in each sample were calculated by linear regression against the calibration curves. Quality control samples (mixed standard solutions at moderate concentrations) were injected every five to 10 samples throughout the analytical sequence, and the relative standard deviation of each target compound in the QC samples was below 15%.

### 2.12 Cell experiments

Human colorectal cancer HT-29 cells and human hepatocarcinoma HepG2 cells were purchased from Pricella Life Science & Technology Co., Ltd. (WuHan, China). HT-29 cells were cultured in Dulbecco’s modified Eagle’s medium (DMEM; Gibco, Grand Island, NY, United States) containing 10% fetal bovine serum (FBS, Excell, Uruguay), 100 U/mL penicillin and 100 μg/mL streptomycin at 37 °C in humidified atmosphere containing 5% CO_2_. HepG2 cells were cultured in MEM containing 10% FBS, 100 μg/mL penicillin and 100 μg/mL streptomycin. LCA (Sigma, Cat. No. L0720800, CAS: 434-13-9) and 3β,7α-dihydroxy-5-cholestenoate (Sigma, Cat. No. 700028P, CAS: 115538-84-6) were mixed at a 1:1 ratio to prepare a bile acid mixture. Cells were seeded in a 96-well plate at 5 × 10^4^ cells/well, incubated with a gradient concentration of bile acid mixture for 24 h, and then cell activity was detected using Cell Counting Kit-8 (CCK-8) kit. Next, cells were seeded in a 12-well plate at 5 × 10^5^ cells/well, and the cells were collected after incubation with a gradient concentration of BAs for 24 h. Total RNA was extracted from cells using a Total RNA Kit (TransGen Biotech, Beijing, China), and reverse-transcribed to cDNA (TAKARA, Beijing, China) according to the manufacturer’s protocol. Quantitative PCR was performed using gene-specific primers and SYBR green PCR Master Mix (TIANGEN BIOTECH, Beijing, China) on a LightCycler^®^480 System (Roche, Switzerland). The housekeeping gene glyceraldehyde-3-phosphate dehydrogenase (GAPDH) was used as an internal control. Primer sequences were listed in Supplementary Table 2. The relative quantitation of gene expression was performed using the comparative Ct (2^-△△Ct^) method, where ΔΔCt represents the difference in Ct values between the target gene and the reference gene, normalized to a control group.

### 2.13 Hepatic glucose output

HepG2 cells were maintained in DMEM medium with 10% FBS. After seeding, the media were replaced with serum-free DMEM for 18 h. After washing, the cells were incubated in glucose-free media supplemented with 10 mM pyruvate and either 100 nM dexamethasone (positive control) or the bile acid mixture (40 μM) for 4 h. Then the cell supernatant was collected for glucose analysis.

### 2.14 Statistical analysis

Data from the GMrepo database were analyzed using the Mann–Whitney *U* non-parametric test for statistical differences between two independent samples. Data from mice and cells are presented as mean ± standard deviation. A normal distribution test was conducted before the *t*-test or one-way analysis of variance (ANOVA). When the data followed a normal distribution, the independent sample *t*-test was used, when the data are not normally distributed, the Mann–Whitney test or Wilcoxon rank-sum test (non-parametric test) was employed. Similarly, the comparison of microbial relative abundance differences and the inter-group differences in α-diversity indices, all use the Wilcoxon rank-sum test. The significance criteria/thresholds for statistical differences are set as follows: ^*^p < 0.05, ^**^p < 0.01, ^***^p < 0.001, ^****^p < 0.0001. Statistical analyses were performed using GraphPad Prism 8.0 (GraphPad Software Inc., San Diego, CA, United States).

## 3 Results

### 3.1 YGMCC0564 attenuated HFD-induced increases in body weight, lipids, and blood glucose in mice

Analysis from the GMrepo database revealed varying abundance of significant Phocaeicola species in fecal samples from healthy and obese individuals. Obese subjects displayed lower *P. dorei* and *P. vulgatus* (Figure 1A and Supplementary Figure 3). Based on our previous research on *P. dorei* YGMCC0564, known for its strong lipid degradation capabilities and favorable safety profile, we designed a study to investigate its impact on attenuating HFD-induced glycolipid metabolic disturbances. In the preliminary dose-range experiment, all three doses significantly attenuated body weight gain relative to the HFD group, no significant differences in body weight were observed among the three YGMCC0564 doses, and 10^9^ CFU/day, which lies within the conventional probiotic dose range, was selected as the dose for the main experiments (Supplementary Figure 4). Male mice were fed a high-fat diet to induce glycolipid metabolic disturbances. Concurrently with HFD feeding (beginning at week 0), mice received daily oral supplementation with YGMCC0564, LGG, or Orlistat for 8 weeks. After 8 weeks on the high-fat diet, the obese mice showed a significant increase in body weight and size (Figure 1B). Notably, intervention with YGMCC0564, LGG, and Orlistat attenuated body weight gain compared to the HFD group. The lipid and glucose metabolism of the mice were disrupted, as evidenced by elevated serum TC and TG (Figures 1C,D), increased LDL and HDL cholesterol (Figures 1E,F), and elevated fasting glucose and insulin resistance (Figures 1G,H) in the HFD group compared with the ND group. Orlistat and LGG marginally decreased TG and fasting glucose but did not significantly improve TC levels, insulin resistance, or LDL and HDL cholesterol. However, YGMCC0564 administration substantially decreased TC, TG, fasting glucose, and insulin resistance, and was the only intervention that significantly lowered serum LDL and HDL cholesterol (Figures 1C–H), demonstrating its efficacy in managing GLMD.

**Figure 1:**
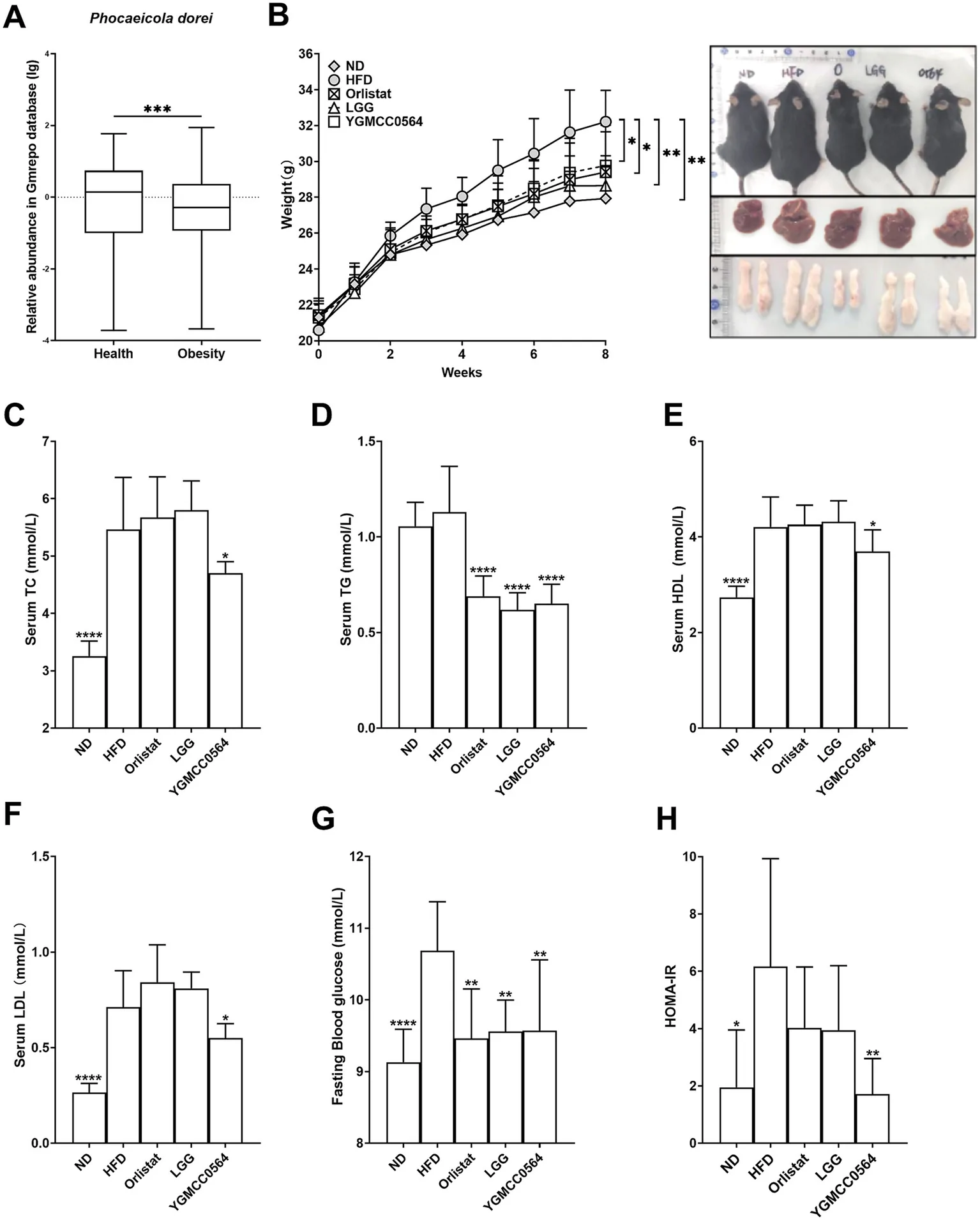
YGMCC0564 attenuated HFD-induced obesity. **(A)** The fecal abundance of *P. dorei* in healthy (*n* = 1,000) and obese (*n* = 541) patients in GMrepo database. **(B)** Average weekly body weight of mice (left) and body size, liver and adipose tissue pictures of mice at week 8 (right). **(C–F)** Serum levels of TC, TG, HDL and LDL. **(G,H)** Fasting blood glucose and homeostasis model assessment of insulin resistance (HOMA-IR) index. Data are presented as mean ± SD. In **(A)**, significance was determined by Mann–Whitney *U* non-parametric test (***p < 0.001). In **(B–H)**, data were from *n* = 10 mice per group, and significance was analyzed by one-way ANOVA followed by Tukey’s multiple comparison test versus the HFD group: *p < 0.05, **p < 0.01, ***p < 0.001, ****p < 0.0001.

### 3.2 YGMCC0564 suppressed hepatic steatosis and gluconeogenesis in obese mice

The HFD group exhibited a significant increase in liver weight and volume (Figure 2B). Although hepatic TG levels showed a trend of increase, this did not reach statistical significance (Figure 2C). Nevertheless, HFD feeding resulted in marked steatosis (*p* < 0.001), as evidenced by excessive fat accumulation and hepatic lobular inflammation (Figure 2A). Pharmacological interventions in the study varied in their effectiveness in reducing liver weight and attenuating steatosis. Supplementation with *P. dorei* YGMCC0564 markedly reduced hepatic TG accumulation, attenuated steatosis, and suppressed inflammatory infiltration, as determined by histopathological scoring of liver sections (*p* < 0.001).

**Figure 2:**
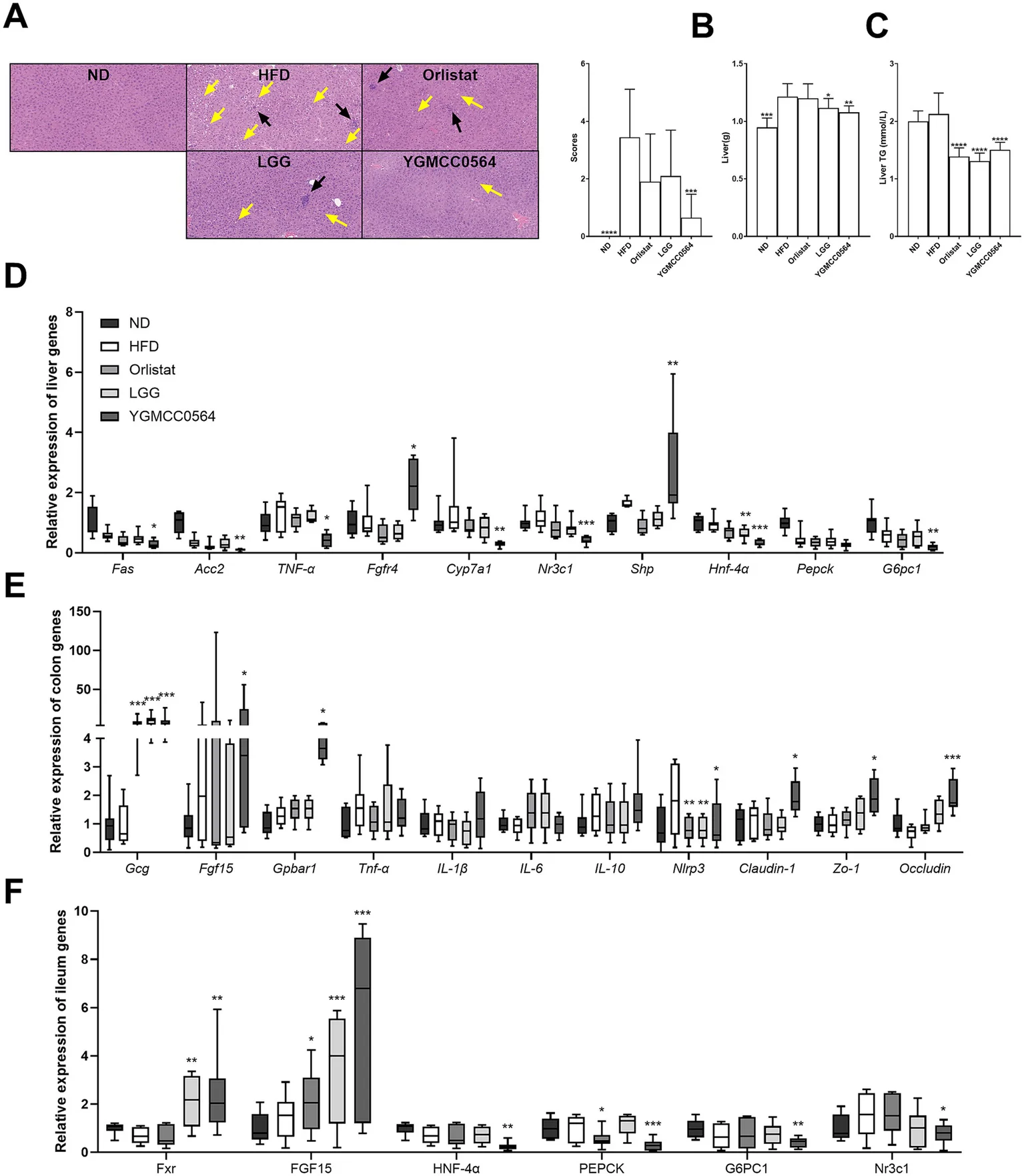
YGMCC0564 suppresses hepatic steatosis in HFD-fed mice. **(A)** Representative photomicrographs of H&E-stained liver sections (left) and quantitative histopathological scoring (right) (20 × magnification; scale bar, 50 μm). Yellow arrows indicate hepatic steatosis; black arrows indicate inflammatory infiltration. **(B)** Liver weights. **(C)** Hepatic triglyceride (TG) level in liver homogenates. **(D–F)** Expression of genes in liver **(D)**, colon **(E)**, and ileum **(F)**. Data are presented as mean ± SD; data were from *n* = 10 mice per group, and significance was analyzed by one-way ANOVA followed by Tukey’s multiple comparison test versus the HFD group: *p < 0.05, **p < 0.01, ***p < 0.001, ****p < 0.0001.

Figure 2D elucidates the protective mechanisms of YGMCC0564: it inhibits hepatic lipogenesis by downregulating *Fasn*, promotes lipid oxidation via *Acc2* downregulation, and decreases the inflammatory marker *Tnfα*, providing comprehensive liver protection. Additionally, our research uncovered an increase in *Fgf15* expression within the colon and ileum (Figures 2E,F). FGF15 is transported to the liver through the gut-liver axis, where it modulates the expression of *Cyp7a1*, *Fgfr4*, and *Shp*. These changes were accompanied by decreases in HNF-4α, PEPCK, and G6pc, key enzymes in gluconeogenesis. Notably, *Nr3c1*, a pivotal gene for hepatic gluconeogenesis encoding the glucocorticoid receptor, showed significant downregulation (Figure 2D). The same gene expression trend was also observed in the ileum, where *Fxr* ascended, while *Hnf-4α*, *Pepck*, *G6pc1* and *Nr3c1* descended (Figure 2F). Meanwhile, after YGMCC0564 intervention, the mRNA expression of *Gpbar1/Tgr5* and barrier proteins (*Claudin-1, Zo-1, Occludin*) in the colon increased, while the expression of the NLRP3 inflammasome decreased (Figure 2E). This effect was specific to the YGMCC0564 intervention group, as Orlistat and LGG did not induce similar changes.

### 3.3 YGMCC0564 promoted the browning process in adipose tissue and reduced inflammation in BAT

In Figure 3A, YGMCC0564 demonstrated a significant decrease in white adipose tissue volume (*p* < 0.05) and reduced lipid accumulation in adipocytes, mirroring the effects of orlistat and LGG (Figure 3B, *P* < 0.001). This finding is consistent with the reported role of FGF15/19 in adipose tissue plasticity and thermogenesis (Morón-Ros et al., 2021). Our study focused on evaluating markers linked to adipocyte browning related to energy expenditure in the YGMCC0564 group. The upregulation of *Fgfr4*, *Ucp1*, *Elovl3*, *Hsl*, *Pparα*, and *Pgc1α* genes (Figure 3C) suggests that YGMCC0564 enhances energy consumption, heat generation in subcutaneous white fat, and promotes browning, potentially through the FGF15-FGFR4-UCP-1 signaling pathway. Additionally, we evaluated key genes within the Gpbar1/TGR5-cAMP-PKA signaling pathway (Figure 3C), including *Tgr5*, *Dio2*, *Adcy1-3*, *Adcy5* and *Creb1*, all of which showed elevated expression levels. Increased expression of FGF21, a known mediator of adipose tissue browning and energy expenditure, was also observed. These findings indicate that YGMCC0564 promotes adipose tissue browning and enhances energy consumption through multiple signaling pathways.

**Figure 3:**
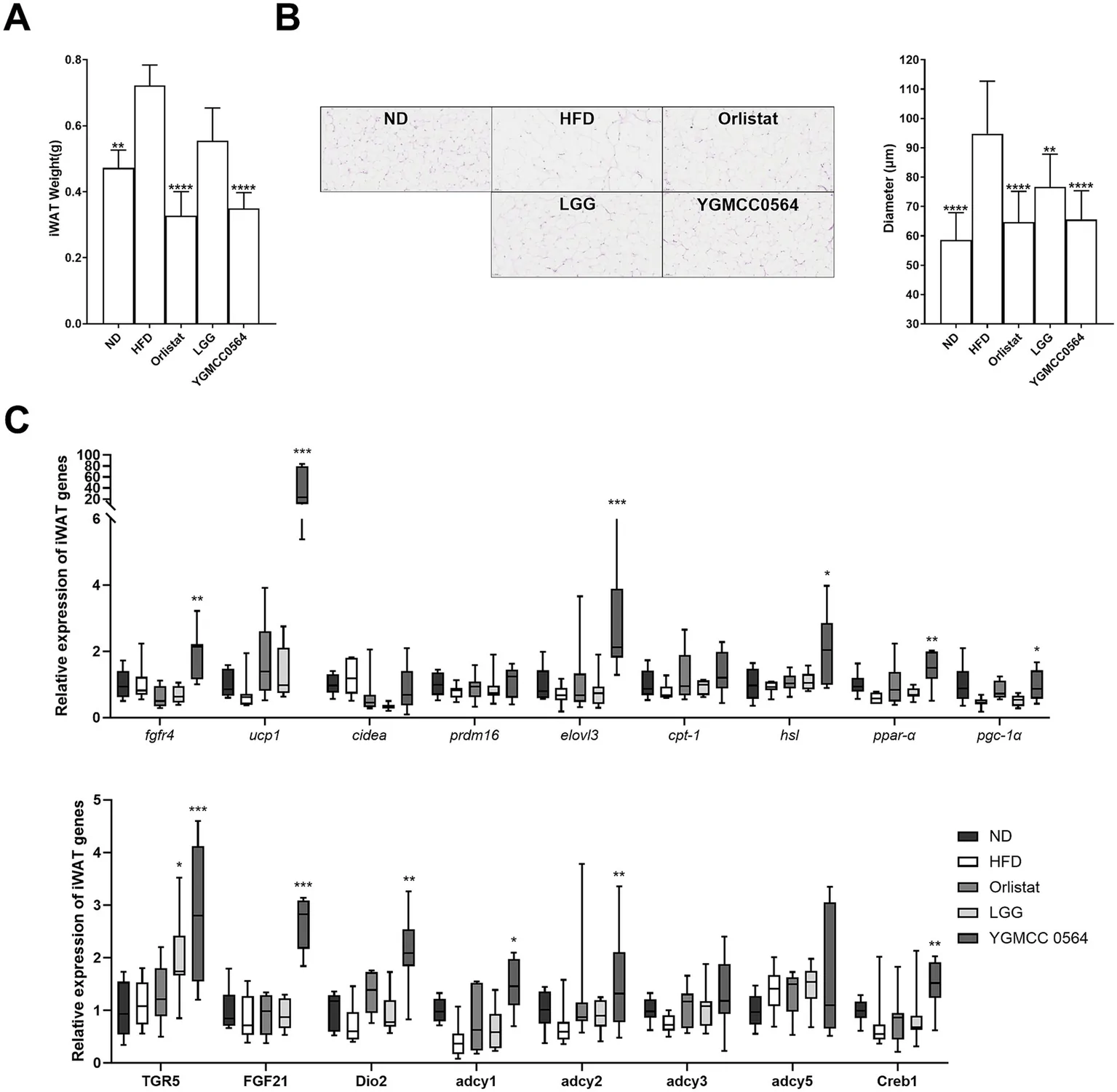
YGMCC0564 intervention promoted the browning process and fatty acid degradation in iWAT. **(A)** Weight of iWAT. **(B)** Representative photomicrographs of H&E-stained iWAT sections (left, 20×; scale bar = 50 μm) and quantification of relative adipocyte diameter (right). **(C)** Expression of genes in iWAT. Data are presented as mean ± SD; data were from *n* = 10 mice per group, and significance was analyzed by one-way ANOVA followed by Tukey’s multiple comparison test versus the HFD group: *p < 0.05, **p < 0.01, ***p < 0.001, ****p < 0.0001.

As shown by H&E staining in Figure 4A, YGMCC0564 intervention attenuated HFD-induced whitening of BAT, decreased intracellular lipid accumulation, and increased brown adipose tissue density (^***^p < 0.001). Moreover, thermogenic genes *Cidea* and *Elovl3* showed significant upregulation in the YGMCC0564 group. HFD-fed mice displayed BAT inflammation, but YGMCC0564 intervention increased *Il4* and *Foxp3* and decreased *Tnfα* and *Il6* (Figure 4B), demonstrating its potent anti-inflammatory properties.

**Figure 4:**
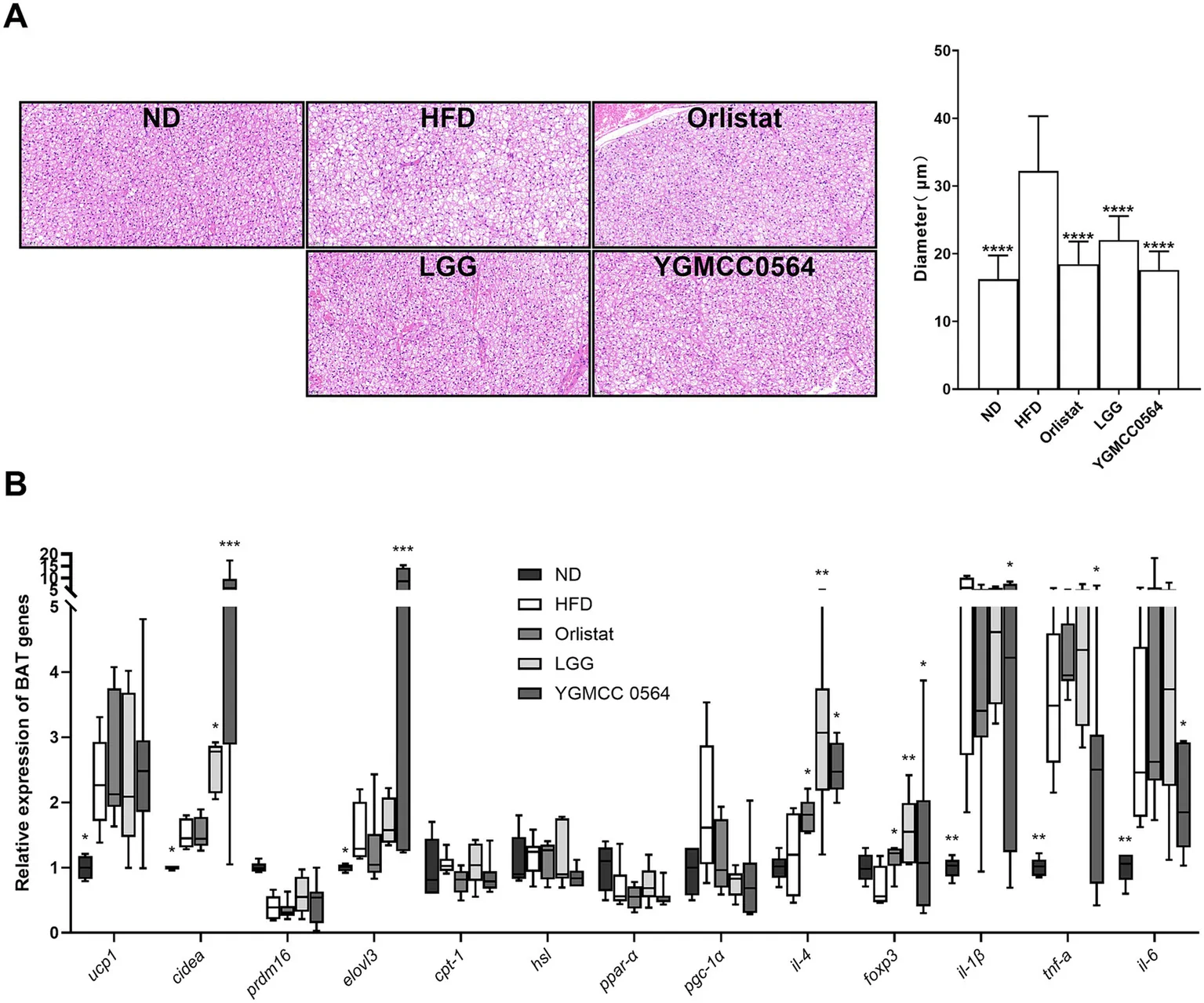
YGMCC0564 intervention activated thermogenic activity and reduced inflammation in BAT. **(A)** Representative images of H&E-stained BAT (left, 20×, scale bar = 50 μm) and relative cell diameter of BAT (right); **(B)** Expression of genes involved in thermogenesis and cytokine in BAT. Data are presented as mean ± SD; data were from *n* = 10 mice per group, and significance was analyzed by one-way ANOVA followed by Tukey’s multiple comparison test versus the HFD group: *p < 0.05, **p < 0.01, ***p < 0.001, ****p < 0.0001.

### 3.4 YGMCC0564 reshaped the gut microbiota and restored short-chain fatty acid production to attenuate HFD-induced weight gain and inflammation in mice

Gut microbiota diversity was assessed using 16S sequencing after administering a high-fat diet. Rarefaction curves reached a plateau (Supplementary Figure 2F), indicating sufficient sequencing depth, and the no-template control included in every PCR run showed no amplification band (Supplementary Figure 1), confirming no reagent contamination. After the 8-week intervention, the abundance of *L. rhamnosus* and *P. dorei* in the intestine increased significantly (Supplementary Figure 2A). The analysis demonstrated a decrease in alpha diversity, assessed by the Shannon index (Supplementary Figure 2B) and Chao index (Supplementary Figure 2C). Orlistat administration decreased both diversity and obesity indices. YGMCC0564 notably influenced beta diversity, shifting the gut microbiota closer to that of the ND group and indicating a healthier microbial composition (*R*^2^ = 0.209, *p* = 0.001, Supplementary Figure 2D). Taxonomic analysis showed that the HFD group had higher relative abundances of *Firmicutes* and *Actinobacteria*, with lower levels of *Bacteroidetes* compared to the ND group. However, YGMCC0564 intervention reduced *Verrucomicrobia* and *Proteobacteria*, while increasing *Firmicutes* and *Actinobacteria* (Supplementary Figure 2E). These findings suggest that YGMCC0564 contributes to regulating the balance of bacterial phyla in the gut microbiota of mice with GLMD.

YGMCC0564 was administered orally to mice, and the fecal copy number of *P. dorei* was significantly higher in the YGMCC0564 group than in the HFD group (Figure 5A). Figure 5B illustrates the genus-level variations between groups, showing that YGMCC0564 intervention induced significant shifts in the abundance of various genera compared to the HFD group. Notably, YGMCC0564 reduced the abundance of *Vampirovibrio*, *Streptococcus*, *Tidjianibacter*, and *Erysipelatoclostridium*, while promoting the abundance of *Lactobacillus*, *unclassified_o_Bacteroidales*, *unclassified_f_Odoribacteraceae*, and *Prevotellamassilia*. The analysis in Figures 5C–E showed that specific genera (*Lactobacillus*, *unclassified_f_Odoribacteraceae*, *unclassified_o_Bacteroidales*, *Limosalactobacillus*, *Duncaniella*, and *Alistipes*) were negatively correlated with obesity markers like body weight, liver weight, iWAT weight, blood lipids (TC, LDL, HDL), and pro-inflammatory cytokines (*Il1β*, *Tnfα*, *Il6*, *Il8*). Conversely, *Desulfovibrio*, *Faecalibaculum*, *Ileibacterium*, *Dubosiella*, *Vampirovibrio*, *Eubacterium*, and *Allobaculum* were associated with unfavorable outcomes, such as increased lipid accumulation and inflammation. *Lactobacillus*, *unclassified_f_Odoribacteraceae*, *unclassified_o_Bacteroidales*, and *Prevotellamassilia* emerged as key contributors to metabolic benefits, highlighting the role of YGMCC0564 in regulating bacterial communities to attenuate HFD-induced weight gain and inflammation.

**Figure 5:**
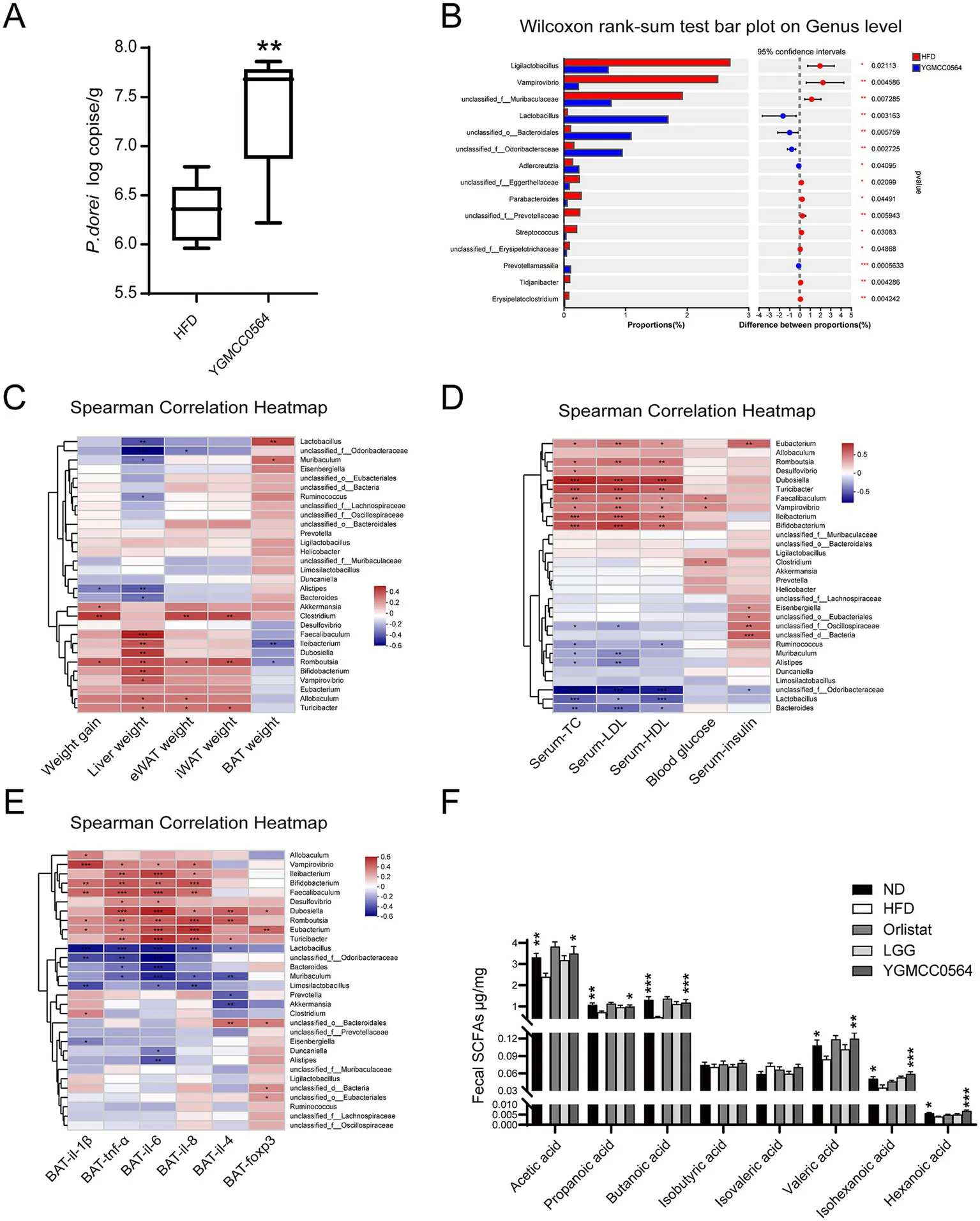
Analysis of intergroup differences in genus levels and intestinal microbial correlation with physiological indications in mice. **(A)** Quantification data from PCR analysis are expressed as log qPCR copy number per g of feces. **(B)** Wilcoxon rank-sum test bar plot at the genus level showing the relative abundance of significantly altered genera between groups. **(C)** Analysis of the correlation between gut microbiota and obesity-related indices. **(D)** Correlations between intestinal microbiota and biochemical indicators. **(E)** Correlations between intestinal microbiota and gene expression in BAT. **(F)** The SCFAs levels in mouse feces quantified by targeted GC–MS.

To further investigate the functional consequences of microbiota remodeling, fecal concentrations of eight SCFAs were quantified by targeted GC–MS with authentic standards (Figure 5F). Of the eight SCFAs quantified, high-fat diet feeding significantly reduced the levels of six, including acetic acid, propionic acid, butyric acid, valeric acid, isohexanoic acid, and hexanoic acid. Notably, YGMCC0564 intervention effectively restored the levels of these SCFAs, bringing them closer to those of the ND group.

### 3.5 YGMCC0564 may attenuate HFD-induced glycolipid metabolism by modulating bile acid levels in mice

YGMCC0564’s impact on GLMD was studied using LC–MS/MS for non-targeted metabolite profiling, revealing clear distinctions between ND and HFD groups via PCA analysis (Figure 6A). The HFD group showed 368 elevated and 659 reduced metabolites compared to ND, whereas the YGMCC0564 group exhibited 518 increased and 90 decreased metabolites relative to the HFD group (Figures 6B,C). KEGG pathway analysis highlighted significant alterations in dopaminergic synapses, bile acid metabolism, and arginine/proline pathways (Figure 6D). YGMCC0564 notably altered fecal metabolites by reducing the secondary bile acid isoLCA, the primary bile acid CDCA, the taurine-conjugated secondary bile acid TDCA, and the secondary bile acid α-muricholic acid (α-MCA), whereas the secondary bile acid LCA and 3β,7α-dihydroxy-5-cholestenoate were enriched in both the ND and YGMCC0564 groups, implying their involvement in these alterations (Figures 6E,F). Integrating metabolomics and microbiomics, *Bacteroides*, *Lactobacillus*, unclassified_f_*Odoribacteraceae*, and *Prevotellamassilia*, which were enriched in the YGMCC0564 group, were found to be associated with the secondary bile acid LCA, 3β,7α-dihydroxy-5-cholestenoate, and the secondary bile acid derivative monoketocholic acid, inversely linked to the primary bile acid CA, the secondary bile acid isoLCA, the secondary bile acid DCA, and the secondary bile acid α-MCA (Supplementary Figure 5A). Pearson correlation analysis revealed positive connections between 3β,7α-dihydroxy-5-cholestenoate and LCA with glucose and lipid homeostasis markers (*Ucp*, *Glp1*, *Fgf15*, *G6pc1*, *Cyp7a1*, and *Shp*), while negatively affecting inflammation and steatosis (Supplementary Figure 5B). These data indicate that LCA and 3β,7α-dihydroxy-5-cholestenoate were putatively identified as key metabolites associated with the metabolic effects of YGMCC0564. The concurrent changes in bile acid levels, microbial taxa, and glucose and lipid homeostasis markers are consistent with a contribution of bile acid metabolism to the attenuation of HFD-induced glycolipid metabolic disturbances.

**Figure 6:**
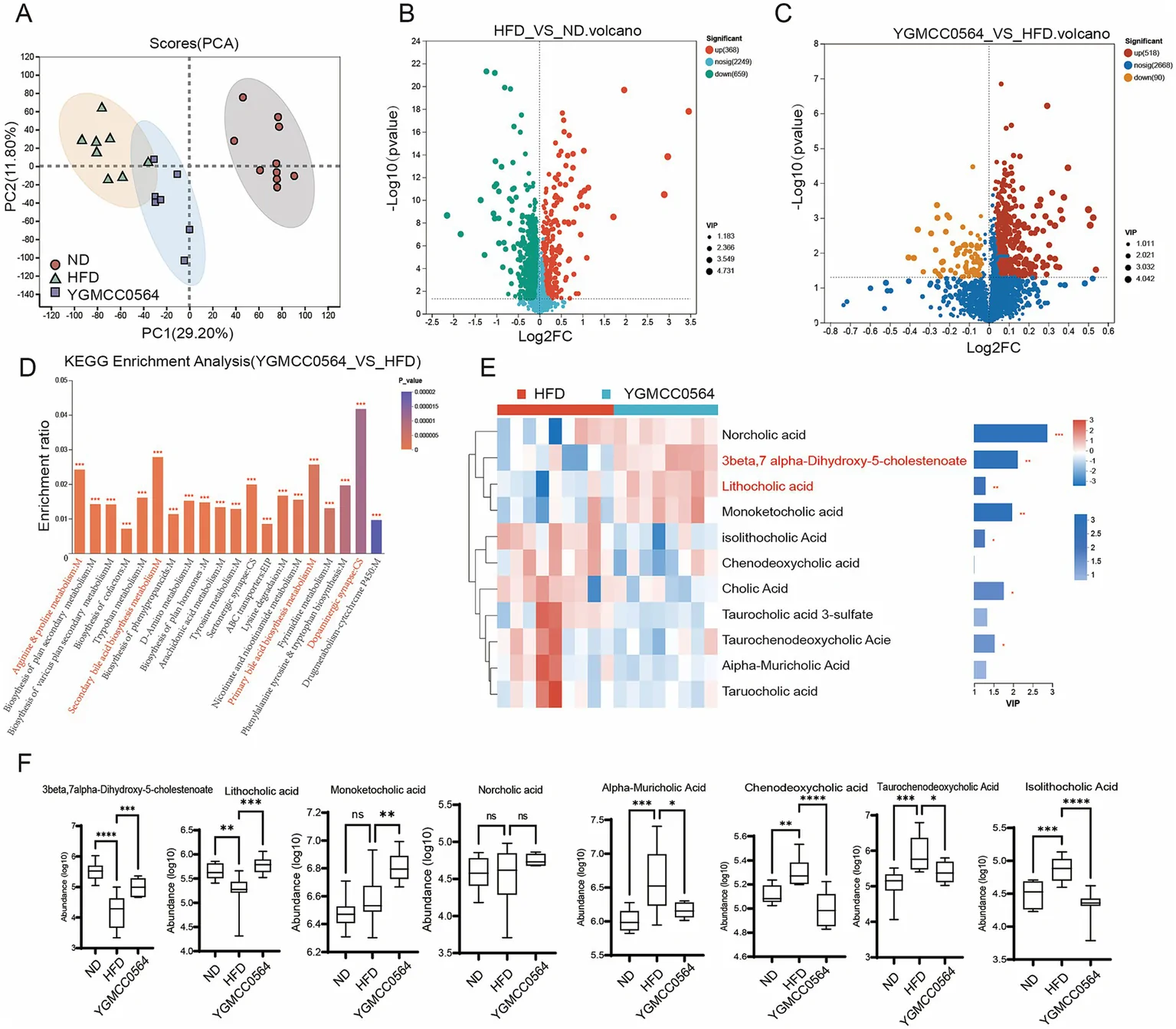
LCA and 3β,7α-dihydroxy-5-cholestenoate were enriched in YGMCC0564 groups. **(A)** β-diversity analysis of the metabolic profiles in colonic contents. **(B)** Volcano plot of differential metabolites resulting from the comparison of the HFD and ND groups. **(C)** Volcano plot of differential metabolites resulting from the comparison of the YGMCC0564 and HFD groups. **(D)** KEGG enrichment analysis from the comparison of the YGMCC0564 and HFD groups. **(E,F)** Differences in bile acids abundance in groups. Error bars represent SD. *p < 0.05, **p < 0.01, ***p < 0.001.

HT-29 and HepG2 cells were exposed to bile acid mixtures to study the effects of LCA and 3β,7α-dihydroxy-5-cholestenoate on glycolipid metabolism. Figure 7 shows no significant cytotoxicity at concentrations up to 40 μM. The FXR-FGF19 signaling pathway was dose-dependently activated, accompanied by increased expression of *Fxr*, *Rxrα*, and *Fgf19* genes in HT-29 cells (Figure 7D). LCA and 3β,7α-dihydroxy-5-cholestenoate were shown to directly activate intestinal FXR-FGF19 signaling. FGF15 is the ortholog of FGF19 in rodents, all of which function by binding to the FGF receptor (FGFR) and α -klotho or β -klotho. Thus, the activation of FXR-FGF19 signaling in human intestinal cells was consistent with the increase in *Fxr* and *Fgf15* in the mouse gut after YGMCC0564 administration. HepG2 cells exhibited activation of the FXR pathway in response to bile acid stimulation, accompanied by the upregulation of *Fxr*, *Rxrα*, and *Shp* genes. Furthermore, through hepatic glucose output assay, we found that glucose production was significantly reduced in HepG2 cells treated with the BA mixture (Figure 7C). Hepatic gluconeogenesis was inhibited (Figure 7E), as reflected by reduced expression of the G6Pase gene in the liver, consistent with observations in YGMCC0564-supplemented mice (Figure 2D).

**Figure 7:**
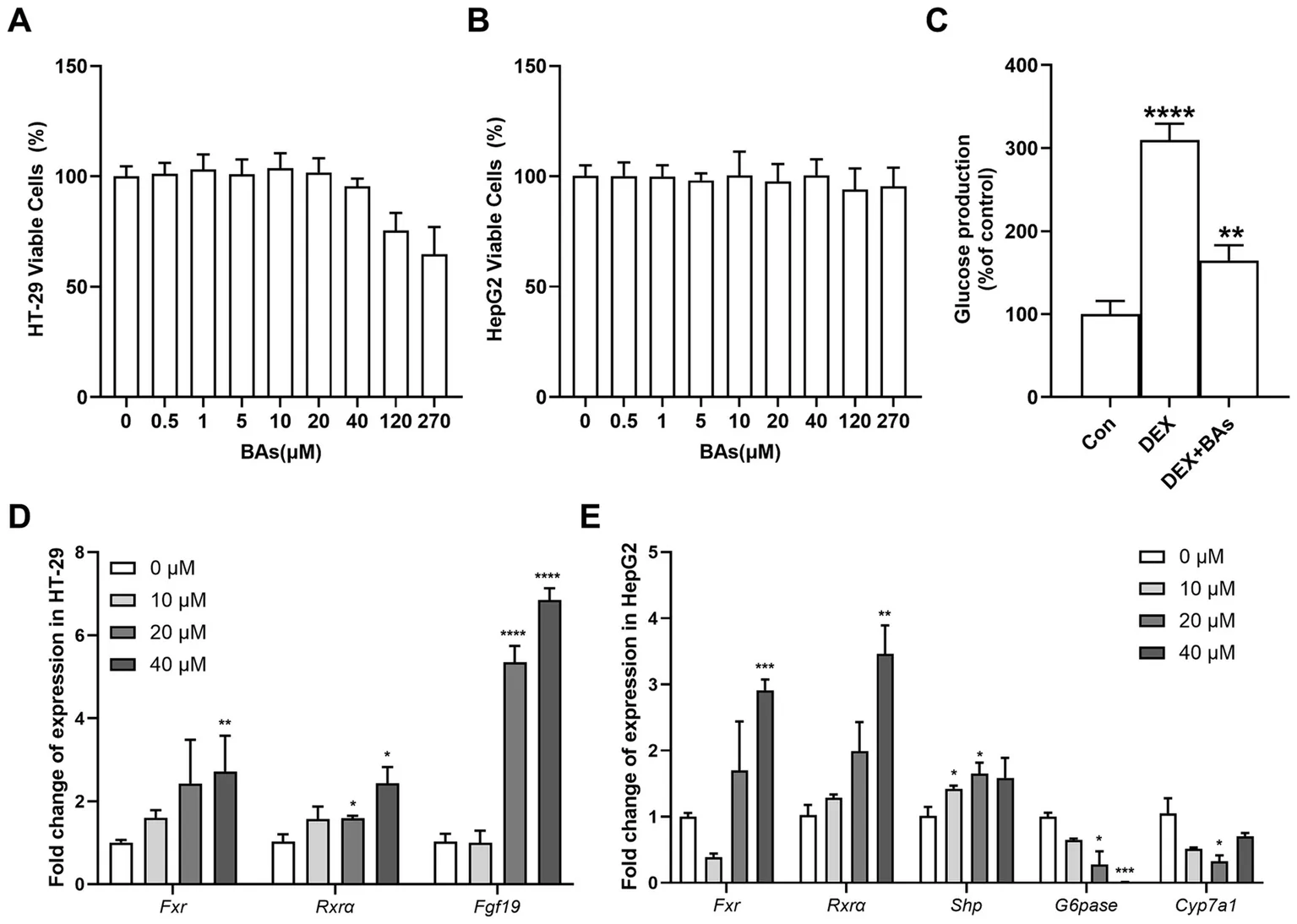
LCA and 3β,7α-dihydroxy-5-cholestenoate can activate FXR-FGF19 signaling in HT-29 and FXR signaling in HepG2. **(A,B)** The CCK-8 cytotoxic effect of the bile acid mixture (LCA: 3β,7α-dihydroxy-5-cholestenoate = 1:1) was evaluated at varying concentrations on HT-29 and HepG2 cells. **(C)** Glucose production assay in HepG2 cells. **(D)** Gene expression was quantified after the HT-29 cells stimulated with the BA mixture for 24 h. **(E)** Gene expression was quantified after the HepG2 cells stimulated with the BA mixture for 24 h. Results are expressed as mean ± standard deviation from three independent experiments. *p < 0.05, **p < 0.01, ***p < 0.001, ****p < 0.0001 compared with the 0 μM group using the one-way ANOVA with Tukey’s Multiple Comparison Test.

### 3.6 Safety of YGMCC0564 in normal mice and nude mice

In our previous studies, *P. dorei* YGMCC0564 demonstrated no hemolytic activity and a low virulence potential (He et al., 2023). To further evaluate its safety, we administered YGMCC0564 acutely to immune-deficient nude mice. No death or weight loss was observed after administration of YGMCC0564 at 1 × 10^9^ CFU/day for 3 days (Figure 8A). Histopathological examination revealed no obvious damage in the colon of nude mice (Figure 8B). The routine blood examination showed no significant differences between the groups (Supplementary Table 3), and the blood biochemical examination also showed no significant differences (Supplementary Table 4). In the chronic toxicity test, YGMCC0564 at a low dose of 1 × 10^9^ CFU/day and a high dose of 1 × 10^10^ CFU/day was orally administered to normal BALB/c mice for 1 month. No significant differences in body weight (Figure 8C) or colon histopathological damage (Figure 8D) were observed. The routine blood examination (Supplementary Table 5) revealed no differences in red blood cell (RBC), hemoglobin (HGB), or platelet (PLT) values between the groups. The blood biochemical examination (Supplementary Table 6) revealed no differences in triglyceride (TG), total cholesterol (TC), alanine aminotransferase (ALT), or aspartate aminotransferase (AST). In conclusion, administration of *P. dorei* YGMCC0564 caused no obvious organ or tissue damage (Figure 9).

**Figure 8:**
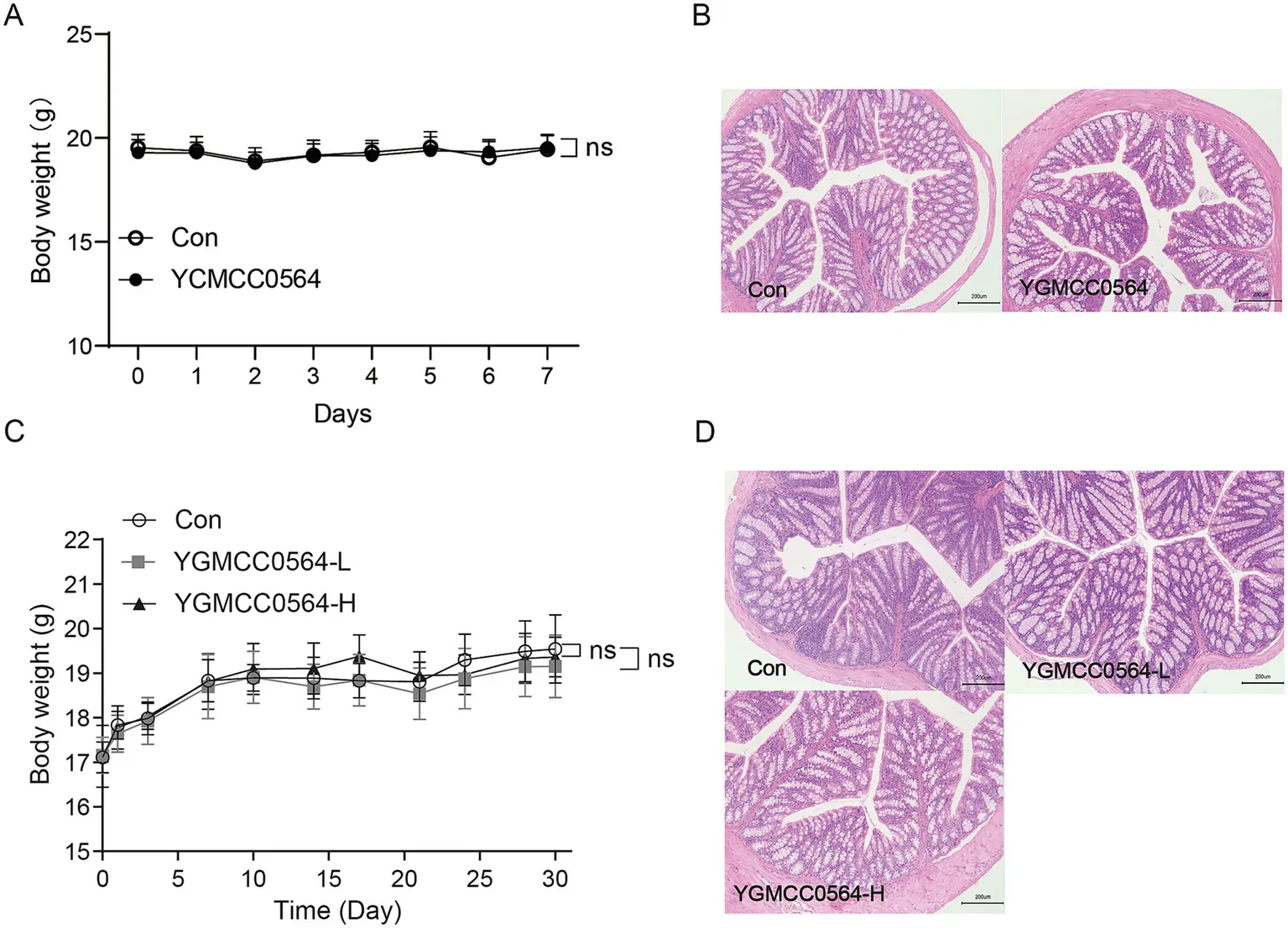
Toxicity of YGMCC0564 to normal mice and nude mice. **(A)** Average daily body weight of nude mice in the acute test. **(B)** Representative images of H&E-stained colon in nude mice (10×, scale bar = 200 μm). **(C)** Body weight of normal mice in the chronic test. **(D)** Representative images of H&E-stained colon in normal mice (10×, scale bar = 200 μm). Data are presented as mean ± SD. *n* = 20 mice per group (10 male and 10 female). Ns (not significant) compared with the control (Con) group using one-way ANOVA with Tukey’s multiple comparison test or Student’s *t*-test.

**Figure 9:**
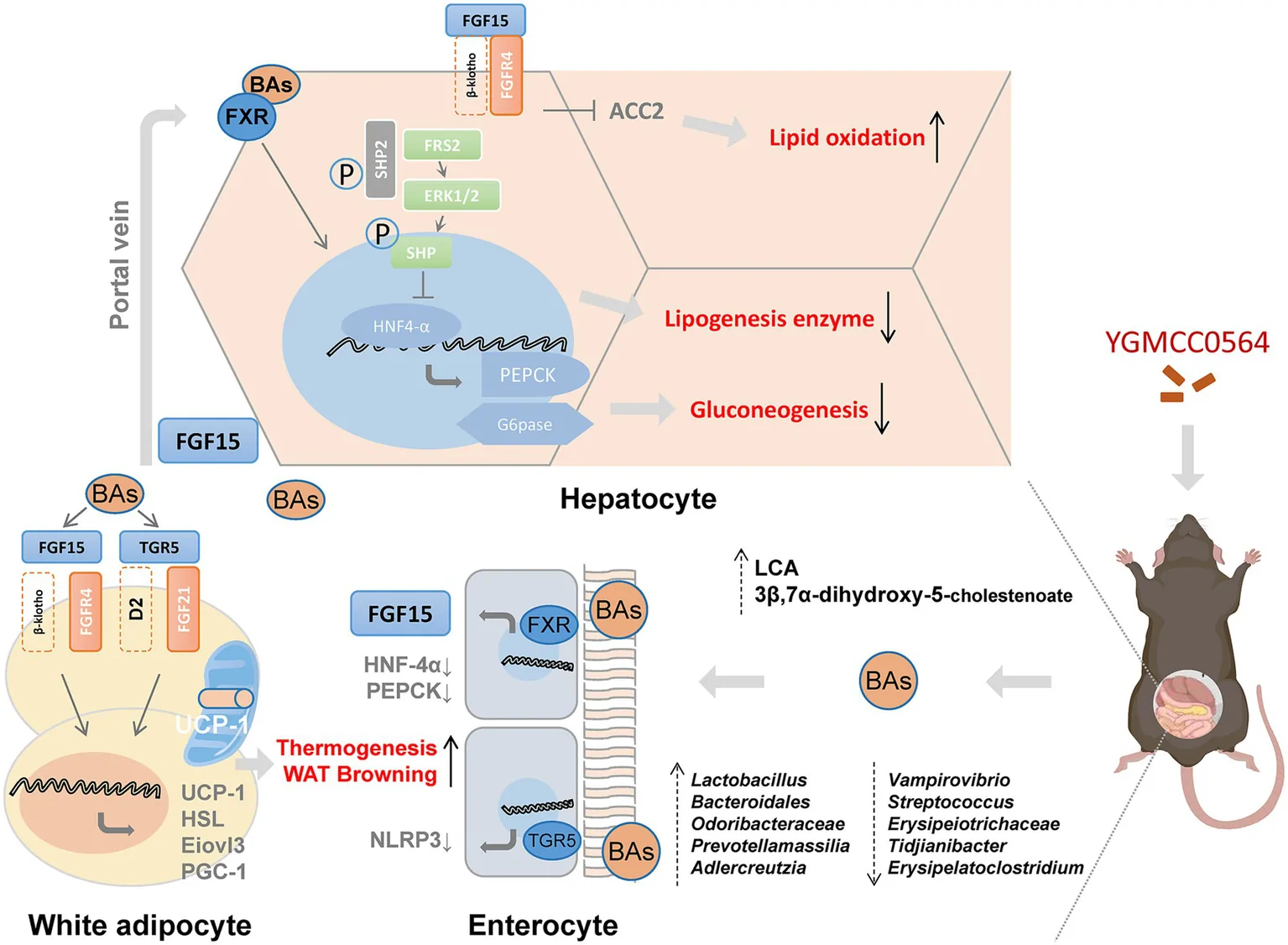
Through its intrinsic BSH activity and gut microbiota remodeling, YGMCC0564 reshapes the intestinal bile acid pool, which may modulate FXR–FGF15/19 and GPBAR1/TGR5 signaling, thereby contributing to the attenuation of HFD-induced hyperglycemia and dyslipidemia.

## 4 Discussion

In this study, *P. dorei* YGMCC0564 attenuated HFD-induced glycolipid metabolic disturbances, and these benefits were accompanied by extensive remodeling of the bile acid (BA) pool and the gut microbiota. Based on integrated multi-omics analysis and *in vitro* functional validation, we propose a stepwise mechanistic framework: (i) YGMCC0564, through its documented BSH activity, deconjugates bile acids and reshapes the intestinal BA pool; (ii) the remodeled BA profile is associated with activation of the FXR–FGF15/19 signaling axis, consistent with the observed modulation of hepatic glycolipid metabolism; (iii) BA-mediated activation of GPBAR1/TGR5 may promote GLP-1 secretion and attenuate intestinal inflammation; and (iv) YGMCC0564-induced microbiota remodeling was accompanied by restored SCFA production, plausibly providing complementary metabolic benefits. Each step is detailed below with the supporting evidence from the present study and the literature.

The most directly evidenced step in the proposed model is BSH-mediated bile acid deconjugation by YGMCC0564. Our previous study confirmed that YGMCC0564 possesses high BSH enzyme activity (He et al., 2023), and co-incubation experiments showed that the strain directly degrades taurodeoxycholic acid (TDCA). This direct action carries significant functional implications. TDCA has been shown to inhibit LCA synthesis by suppressing the bai operon in intestinal bacteria (Chen et al., 2024), and YGMCC0564-mediated TDCA degradation would therefore be expected to relieve this suppression. Consistent with this prediction, *in vivo* bile acid profiling revealed that YGMCC0564 intervention decreased TDCA, increased LCA, elevated 3β,7α-dihydroxy-5-cholestenoate and monoketocholic acid, and reduced α-MCA. With this qualification, the convergence of *in vitro* causal evidence (direct TDCA degradation) and *in vivo* observational data identifies bile acid remodeling as the plausible initiating event of the proposed model. The overall remodeling of the *in vivo* bile acid profile therefore likely reflects the combined effects of the direct enzymatic activity of the administered strain and the secondary remodeling of the gut microbial ecosystem. This BSH-driven BA modulation also aligns with broader evidence that microbiota-mediated bile acid metabolism plays a pivotal role in maintaining glycaemic and lipid homeostasis (Funabashi et al., 2020; Huang et al., 2019; Jia et al., 2017).

The remodeled BA pool provides a plausible mechanistic basis for enhanced FXR signaling. Among the bile acids elevated by YGMCC0564, LCA is a known FXR agonist, and 3β,7α-dihydroxy-5-cholestenoate may also contribute to receptor activation. FXR, a key bile acid receptor, governs lipid and glucose balance by regulating bile acid synthesis, lipid metabolism, and glucose breakdown (Claudel et al., 2005). In our study, YGMCC0564 intervention upregulated colonic FXR and FGF15 expression. Importantly, the functional capacity of the elevated bile acids was directly supported *in vitro* (Figure 7D). Our *in vivo* observations nevertheless align with prior reports that intestinal FXR activation enhances LCA production and improves insulin sensitivity (Fang et al., 2015; Pathak et al., 2018). Downstream of FXR, FGF15/19 acts on the liver to inhibit bile acid and glucose synthesis while promoting glycogen and protein synthesis (Gadaleta and Moschetta, 2019; Inagaki et al., 2005; Kir et al., 2011). At the molecular level, FGF19 has been shown to lower triglyceride (TG) levels through SHP phosphorylation–mediated STAT3 activation and PGC-1 suppression, thereby inhibiting adipogenic enzyme production (Bhatnagar et al., 2009; Fu et al., 2004; Kim et al., 2023), and to induce white adipose browning via UCP1 and Dio2 upregulation (Morón-Ros et al., 2021). Our finding that YGMCC0564 upregulated intestinal FGF15 is therefore consistent with these established downstream mechanisms. Regarding the suppressed hepatic gluconeogenic gene expression observed *in vivo*, both an indirect FGF15–FGFR4 enterohepatic mechanism and a direct hepatic action of circulating bile acids are plausible and are not mutually exclusive, and the *in vitro* HepG2 data (Figures 7C,E) support the possibility of a direct hepatic contribution. The present study cannot discriminate between the direct hepatic action of bile acids and the indirect FGF15-mediated pathway. The precise contribution of the FXR-FGF15 axis to the overall metabolic effects of YGMCC0564 requires further causal validation (Huang et al., 2019; Kubota et al., 2011).

In parallel with FXR activation, the remodeled BA profile may also activate GPBAR1/TGR5, another major bile acid receptor expressed in enteroendocrine L cells (Shapiro et al., 2018; Sorrentino et al., 2020). In YGMCC0564-supplemented mice, both GLP-1 and GPBAR1/TGR5 expression in the colon increased significantly, suggesting enhanced TGR5 signaling. Upon activation, GPBAR1/TGR5 stimulates GLP-1 secretion via the cAMP-PKA signaling pathway (Gao et al., 2026; Liu et al., 2024), thereby improving insulin sensitivity and glucose homeostasis. GLP-1, encoded by the *Gcg* gene, is an enteroendocrine hormone that improves glycaemic control and attenuates inflammation (Fan and Pedersen, 2020). Additionally, TGR5 activation inhibits NF-κB and Nlrp3-mediated inflammatory pathways, reducing the release of pro-inflammatory cytokines including TNF-α, IL-6, and IL-1β (Liu et al., 2024; Sun et al., 2025; Yan et al., 2025), which may collectively contribute to the attenuated inflammatory responses observed in this study. In future studies, we plan to employ a targeted LC–MS/MS approach with certified reference standards to perform absolute quantification of key bile acids, including LCA and 3β,7α-dihydroxy-5-cholestenoate, in feces, portal vein serum, liver, and ileal contents, and to determine their concentration gradients across the gut–liver axis.

Beyond bile acid signaling, YGMCC0564-induced gut microbiota remodeling was accompanied by restoration of fecal SCFA production, plausibly providing an additional metabolic regulatory layer. Multi-omics analysis revealed that YGMCC0564 promoted beneficial bacteria, including *Bacteroidetes*, *Lactobacillus*, *unclassified_f_Odoribacteraceae*, and *Prevotellamassilia*. Consistent with this microbiota shift, targeted GC–MS quantification showed that high-fat diet reduced fecal SCFA concentrations, while YGMCC0564 intervention restored acetic, propionic, and butyric acid levels. *P. dorei* is known to generate propionate, butyric acid, and acetic acid (He et al., 2023), suggesting that the observed SCFA restoration may arise from both direct bacterial production and indirect microbiota remodeling. SCFAs, particularly butyric and propionic acids, inhibit adipose tissue growth via G protein-coupled receptor 43 (GPR43) and promote leptin production (Chambers et al., 2014; Kimura et al., 2013; Xiong et al., 2004), and can stimulate GLP-1 secretion from enteroendocrine L cells, potentially synergizing with the bile acid-mediated GLP-1 pathway described above.

Orlistat is a clinical drug for weight loss (Jagtap and Paul, 2025). It reduces fat absorption by inhibiting the activity of pancreatic lipase (Chianelli et al., 2024). Its mechanism of action is often associated with gastrointestinal adverse reactions, including abdominal pain, bloating, diarrhea, oily stools, and it may also affect the absorption of fat-soluble vitamins (Benedictus et al., 2025). In our study results, orlistat significantly reduced the volume of white adipose tissue, with an effect superior to that of probiotics. However, it had no alleviating effect on liver volume and inflammation, and even showed an increase in the inflammatory factor *Il6* in brown adipose tissue. Compared with the probiotic intervention groups, the gut microbiota structure after orlistat intervention was further away from that of the normal diet group, and the abundance of *Firmicutes* increased significantly, even exceeding that of the HFD group. These findings indicate that although orlistat effectively inhibits fat absorption, it also disrupts the gut microbiota. *Lactobacillus rhamnosus* GG, as a first-generation probiotic, performed exceptionally well in reducing body weight. However, compared with YGMCC0564, it showed weaker effects on reducing blood lipids, alleviating insulin resistance, reducing liver fat, alleviating liver injury, and attenuating inflammation. Together, within the endpoints and conditions examined in this study, YGMCC0564 attenuated HFD-induced symptoms, with an overall profile that compared favorably with orlistat and the first-generation probiotic LGG at multiple metabolic endpoints.

The safety assessment of YGMCC0564 in the present study was preliminary and restricted to an acute test (3-day dosing followed by 7-day monitoring) and a 4-week subchronic toxicity test in mice. This scope does not constitute a comprehensive systemic toxicological evaluation. Broader investigations, such as longer-term studies, histopathological examination of additional organs, genotoxicity assays, and reproductive or developmental toxicity tests, are still warranted before any clinical translation.

Several limitations of the current study should be acknowledged. First, while the direct BSH-mediated degradation of TDCA by YGMCC0564 was experimentally demonstrated through co-incubation, the subsequent steps in the proposed mechanism, including BA-mediated activation of FXR and TGR5, downstream hepatic signaling, and SCFA-mediated metabolic effects, are primarily supported by correlative multi-omics data and established pathway knowledge rather than direct causal evidence. The expression changes observed (e.g., upregulation of FXR, FGF15, TGR5, GLP-1) are consistent with pathway activation but do not constitute proof of causality, and the relative contribution of each mechanism (FXR-FGF15/19 vs. TGR5-GLP-1 vs. SCFA-GPR43) remains unclear. Future studies employing receptor knockout models (e.g., FXR^−/−^ or TGR5^−/−^ mice), pharmacological receptor antagonists, gnotobiotic animal models, fecal microbiota transplantation, or antibiotic-depletion rescue assays would be necessary to definitively establish the causal contributions of each pathway. Direct evidence of *in situ* expression of key functional enzymes such as BSH, or of *in situ* production of relevant metabolites by the strain within the intestinal environment, is not yet available, and closing this gap will require emerging *in situ* tools. Second, the bile acid data are derived from non-targeted metabolomics and are expressed as relative abundances, and absolute quantification of key bile acids using certified standards has not yet been performed; moreover, because multiple bile acid species changed in opposite directions, the net agonist or antagonist activity of the total intestinal bile acid pool toward FXR and TGR5 remains to be determined, which limits inferences regarding concentration-dependent receptor activation. Third, intestinal barrier integrity was inferred from tight junction gene expression only, and functional permeability assays or protein-level validation were not performed. Fourth, efficacy was assessed exclusively under HFD challenge and at a single dose (10^9^ CFU/day), and the 4-week subchronic toxicity study in normal chow-fed mice informs tolerability rather than efficacy in normal-diet animals. Finally, individual variations in gut microbiota composition, dosage effects, and long-term safety profiles need to be evaluated before clinical translation. Despite these limitations, our findings provide a multi-omics-based framework for understanding how P. dorei YGMCC0564 modulates glycolipid metabolism and highlight the preventive potential of BSH-active probiotics in attenuating diet-induced metabolic disturbances.

In addition, it should be emphasized that the present study employed a prevention design, in which YGMCC0564 supplementation was administered concurrently with HFD feeding rather than after the establishment of obesity. Consequently, the results demonstrate that YGMCC0564 can attenuate the development of HFD-induced weight gain, dyslipidemia, insulin resistance, and hepatic steatosis, but they do not address whether YGMCC0564 can reverse these conditions once they are fully established. A treatment-paradigm study, in which mice are first rendered obese and then receive YGMCC0564, would be necessary to evaluate the therapeutic efficacy of this strain.

## 5 Conclusion

In this prevention-design study in HFD-fed mice, *P. dorei* YGMCC0564 attenuated HFD-induced glycolipid metabolic disturbances, and these benefits may be achieved through two complementary gut-liver signaling pathways. One pathway couples the remodeled fecal bile acid profile to intestinal FXR-FGF15/19 signaling and reduced hepatic gluconeogenic gene expression, and the two bile acids elevated by YGMCC0564 directly activated FXR-FGF19 signaling and suppressed gluconeogenic gene expression and glucose output *in vitro*. The other couples YGMCC0564-induced microbiota remodeling and restored SCFA levels to colonic GPBAR1/TGR5-GLP-1 signaling, attenuated adipose tissue inflammation, and improved glucose and lipid homeostasis. Within the tested conditions, doses, and durations, YGMCC0564 showed no observable adverse effects and compared favorably with the first-generation probiotic LGG and the anti-obesity drug orlistat at multiple metabolic endpoints. To our knowledge, this is the first prevention-design study to connect a *P. dorei* strain with modulation of these two gut-liver signaling axes, and the integration of *in vivo* multi-omics with *in vitro* functional validation establishes a testable framework for microbiota-directed intervention on the gut-liver axis, positioning YGMCC0564 as a next-generation probiotic candidate for the prevention of diet-induced obesity, insulin resistance, hepatic steatosis, and related NAFLD/MASLD conditions.

## Data Availability

The raw data supporting the conclusions of this article will be made available by the authors, without undue reservation.

## Footnotes

1
https://www.r-project.org

2
https://www.rstudio.com

3
http://rdp.cme.msu.edu
