# Supplementary material for: Phocaeicola dorei YGMCC0564 attenuates HFD-induced glycolipid metabolic disorders in association with remodeling of the gut microbiota and bile acid profiles
Source: Front Microbiol. 2026 Sep 21;17:1911556. doi: 10.3389/fmicb.2026.1911556 (PMC13637672; doi:10.3389/fmicb.2026.1911556)
Supplement: Supplementary file 1 [file Supplementary_file_1.DOCX]

Figures S1-S5 and Table S1-7 (see below)

**Supplementary Figure 1**.Agarose gel electrophoresis of the PCR amplicons (bacterial 16S rRNA gene V3-V4 region). M, DL2000 DNA marker. CK, no-template control (blank control) included in every PCR run, shows no amplification band. Lanes 1-50 are the 50 study samples analyzed in this work, and lanes 51-70 are samples from other batches not included in this study.

**Supplementary Figure 2**. YGMCC0564 altered gut microbiota diversity and composition in HFD-fed mice. (A)Abundance of *L. rhamnosus* and *P. dorei* at baseline and week 8. (B-C) Shannon and Chao indices in alpha-diversity analysis. (D) PCoA plot analysis from each sample. (E) Bacterial community at the phylum level. (F) Rarefaction curves of observed operational taxonomic units (sobs index), indicating sufficient sequencing depth. Data are presented as mean ± SD. Data were from n = 10 mice per group.

**Supplementary Figure 3**. Relative abundance of *Phocaeicola spp.* in the intestinal tract of the population.(A-B) Relative abundance of *P. vulgatus* and *P. massiliensis* in the intestinal tract of the population. (C) Comparison of relative abundance for major Phocaeicola species between healthy and obese populations. Data are presented as mean ± SD. Significance was determined by an unpaired two-tailed Student’s *t*-test (*****p* < 0.0001, ns > 0.05).

**Supplementary Figure 4.** Average weekly body weight of mice orally gavaged with 10^8^, 10^9^, or 10^10^ CFU of YGMCC0564 over an 8‑week period. data were from n = 8 mice per group, and significance was analyzed by one-way ANOVA followed by Tukey’s multiple comparison test versus the HFD group: **p* < 0.05, ***p* < 0.01, ****p* < 0.001, *****p* < 0.0001.

**Supplementary Figure 5**. Bile acids in colonic contents were closely related to intestinal flora and indications of glucose and lipid metabolism. (A) Network diagram of correlation between bile acids and intestinal flora; (B) Heat map correlation between bile acids and indications of glucose and lipid metabolism.

**Supplementary Table 1.** Composition of the high-fat diet used in the study (fat providing 48.5% of total calories)

| **Component** | **gm%** | | **kcal%** |
| --- | --- | --- | --- |
| Protein | 24.20% | | 19.80% |
| Carbohydrate | 40.10% | | 31.70% |
| Fat | 27.40% | | 48.50% |
| Total | | 100 | |
| kcal/gm | | 4.7 | |
| Additional Components | | wt% | |
| Crude fiber | | 5.80% | |
| Calcium | | 1.70% | |
| Phosphate | | 1.10% | |
| Lysine | | 1.70% | |
| Methionine + Cystine | | 0.80% | |
| Arginine | | 1.30% | |
| Histidine | | 0.90% | |
| Tryptophan | | 0.40% | |
| Phenylalanine | | 1.60% | |
| Thonine | | 0.60% | |
| Leucine | | 1.60% | |
| Isoeucine | | 1.40% | |
| Valine | | 1.60% | |
| Magnesium | | 0.23% | |
| Potassium | | 0.40% | |
| Sodium | | 0.20% | |
| Vitamin A | | 13000IU / Kg | |
| Vitamin D | | 1800IU / Kg | |
| Vitamin E | | 140IU / Kg | |
| Vitamin K | | 7 IU / Kg | |
| Choline | | 1360mg / Kg | |
| Iron | | 132 mg / Kg | |
| Manganese | | 85 mg / Kg | |
| Copper | | 14 mg / Kg | |
| Zinc | | 31 mg / Kg | |

Data are presented as percentage by weight (gm%), percentage of total calories (kcal%), or absolute content per kilogram of feed as indicated.

| **Supplementary Table 2.** Sequences of primers used for quantitative real-time PCR (qPCR) analysis in mouse tissues and human cells. | | | |
| --- | --- | --- | --- |
| **Gen Name** | **Forward primer (5’-3’)** | **Reverse primer (5’-3’)** | **efficiencies(%)** |
| Mouse Tissue | | | |
| m-*β-actin* | CTGCGTTTTACACCCTTTCTTTG | GCCATGCCAATGTTGTCTCTTAT | 103.86 |
| m-*Fas* | TGCTCCCAGCTGCAGGC | GCCCGGTAGCTCTGGGTGTA | 112.86 |
| m-*Srebp2* | ACGCAAAGGTCAAGGCTG | AGCTTCACAAAGACGCTCA | 97.93 |
| m-*Tnf-α* | TGATCCGCGACGTGGAA | ACCGCCTGGAGTTCTGGAA | 93.46 |
| m-*Glp-1(Gcg)* | CCGCCGTGCCCAAGA | CATCATGACGTTTGGCAATGTT | 114.9 |
| m-*Pyy* | ACGGTCGCAATGCTGCTAAT | GACATCTCTTTTTCCATACCGCT | 119.76 |
| m-*Fgf15* | GCCATCAAGGACGTCAGCA | CTTCCTCCGAGTAGCGAATCAG | 93.32 |
| m-*Glp-1r* | TCAGAGACGGTGCAGAAATG | CAGCTGACATTCACGAAGGA | 98.09 |
| m-*Cyp7a1* | AGCAATGAAAGCAGCCTCTGA | TGATGCTATCTAGTACTGGCAGGT | 97.72 |
| m-*Nr3c1* | CCGGGTCCCCAGGTAAAGA | TGTCCGGTAAAATAAGAGGCTTG | 93.66 |
| m-*Hnf-4α* | GGTCAAGCTACGAGGACAGC | ATGTACTTGGCCCACTCGAC | 109.49 |
| m-*Pepck* | CTGCATAACGGTCTGGACTTC | CAGCAACTGCCCGTACTCC | 97.58 |
| m-*G6pc1* | CGACTCGCTATCTCCAAGTGA | GTTGAACCAGTCTCCGACCA | 106.39 |
| m-*Il-27ra* | CACCAGAGTCAGAAATACCATCCCA | CCAGATGAGGAGTTTGTCAGCCATG | 95.52 |
| m-*Tgr5* | CTGTGTGAGATCCGCCGAC | CGACGCTCATAGGCCAAGA | 119.02 |
| m-*Ucp1* | CTTTGCCTCACTCAGGATTGG | ACTGCCACACCTCCAGTCATT | 98.93 |
| m-*Cidea* | TGCTCTTCTGTATCGCCCAGT | GCCGTGTTAAGGAATCTGCTG | 93.59 |
| m-*Prdm16* | AATGCTGTGGATGCCTGACA | CTCGTCCCCCTTCTGTCCTA | 113.4 |
| m-*Elovl3* | TCCGCGTTCTCATGTAGGTCT | GGACCTGATGCAACCCTATGA | 108.49 |
| m-*Cpt1* | ATGTATCGCCGCAAACTGGACC | CTCTGAGAGGTGCTGTAGCAAG | 105.89 |
| m-*Hsl* | CCAGCCTGAGGGCTTACTG | CTCCATTGACTGTGACATCTCG | 103.87 |
| m-*Ppar-α* | TTCTTGCGATACACTCTGGTGC | CGACAGACAGGCACTTGTGAAA | 99.29 |
| m-*Pgc-1α* | AGCCGTGACCACTGACAACGAG | GCTGCATGGTTCTGAGTGCTAAG | 117.92 |
| m-*Fxr* | CCCCTGCTTGATGTGCTAC | CGTGGTGATGGTTGAATGTC | 104.11 |
| m-*Shp* | TCTGCAGGTCGTCCGACTATTC | AGGCAGTGGCTGTGAGATGC | 101.87 |
| m-*Retn* | TGGAGTGCCAGAGCGTCACCT | ACTGGCAGTGACATGTGGTCTC | 119.18 |
| m-*Leptin* | TTCACACACGCAGTCGGTAT | GCTGGTGAGGACCTGTTGAT | 118.93 |
| m-*Il-4* | GGTCTCAACCCCCAGCTAGT | GCCGATGATCTCTCTCAAGTGAT | 112.58 |
| m-*Foxp3* | ACCATGACTAGGGGCACTGTA | TCCACTCGCACAAAGCACTT | 107.42 |
| m-*Il-1β* | CAGGCAGGCAGTATCACTCATTG | CGTCACACACCAGCAGGTTATC | 96.25 |
| m-*Il-6* | CCAGTTGCCTTCTTGGGACT | GGTCTGTTGGGAGTGGTAT | 118.7 |
| Probiotics | | | |
| p-LGG-1 | CTCGGCAATGGCATCAAGTT | GGCATCGTTGGCACAGTATT | 118.57 |
| *BD_0416(B.D)* | TAGGCGCCGATTGGAAAACT | AGTCCCACTACGCCAATGTC | 101.34 |
| Human Cells | | | |
| *GAPDH* | GATTCCACCCATGGCAAATTC | CTGGAAGATGGTGATGGGATT | 113.17 |
| h-*Fxr* | AACCATACTCGCAATACAGCAA | ACAGCTCATCCCCTTTGATCC | 107.48 |
| h-*Fgf19* | CGGAGGAAGACTGTGCTTTCG | CTCGGATCGGTACACATTGTAG | 110.36 |
| h-*Rxrα* | GACGGAGCTTGTGTCCAAGAT | AGTCAGGGTTAAAGAGGACGAT | 94.52 |
| h-*Shp2* | CCCCAAGGAATATGCCTGCC | TAGGGCGAAAGAAGAGGTCCC | 92.21 |
| h-*G6Pase* | CTACTACAGCAACACTTCCGTG | GGTCGGCTTTATCTTTCCCTGA | 117.67 |
| h-*Cyp7a1* | AGAAGCATTGACCCGATGGAT | AGCGGTCTTTGAGTTAGAGGA | 107.78 |

Gene symbols are italicized. Abbreviations: m, mouse;p,probiotics; h, human.

| **Supplementary Table 3.** Blood routine examination of nude mice after 7-day oral administration with 10⁹ CFU/day of YGMCC0564. | | | |
| --- | --- | --- | --- |
| **Parameter** | **Unit** | **Con** | **YGMCC0564** |
| WBC | 10^9^/L | 5.95±2.18 | 6.15±1.50 |
| Neu# | 10^9^/L | 1.20±0.38 | 1.512±0.43 |
| Lym# | 10^9^/L | 4.31±1.88 | 4.088±1.26 |
| Mon# | 10^9^/L | 0.23±0.11 | 0.355±0.21 |
| Eos# | 10^9^/L | 0.19±0.09 | 0.173±0.04 |
| Bas# | 10^9^/L | 0.02±0.01 | 0.022±0.01 |
| Neu% | % | 21.23±6.20 | 25.10±5.72 |
| Lym% | % | 70.59±8.19 | 65.51±8.29 |
| Mon% | % | 4.03±2.09 | 6.09±4.65 |
| Eos% | % | 3.83±2.97 | 2.91±0.57 |
| Bas% | % | 0.31±0.17 | 0.39±0.17 |
| RBC | 10^12^/L | 10.86±1.71 | 11.40±0.42 |
| HGB | g/L | 183.78±28.24 | 190.00±7.23 |
| HCT | % | 57.37±9.92 | 62.52±2.52 |
| MCV | fL | 55.04±2.37 | 54.87±2.22 |
| MCH | pg | 16.60±0.53 | 16.66±0.28 |
| MCHC | g/L | 301.78±12.2 | 304.10±10.94 |
| RDW-CV | % | 17.83±4.51 | 21.38±7.78 |
| RDW-SD | fL | 39.22±11.6 | 47.03±18.11 |
| PLT | 10^9^/L | 822.56±249.82 | 947.10±100.91 |
| MPV | fL | 7.46±0.98 | 6.83±0.66 |
| PDW |  | 16.52±0.58 | 16.50±0.27 |
| PCT | % | 0.50±0.14 | 0.65±0.11 |

Abbreviations: WBC, white blood cells; Neu, neutrophils; Lym, lymphocytes; Mon, monocytes; Eos, eosinophils; Bas, basophils; RBC, red blood cells; HGB, hemoglobin; HCT, hematocrit; MCV, mean corpuscular volume; MCH, mean corpuscular hemoglobin; MCHC, mean corpuscular hemoglobin concentration; RDW, red cell distribution width; PLT, platelets; MPV, mean platelet volume; PDW, platelet distribution width; PCT, plateletcrit.Data are presented as mean ± standard deviation (SD).

| **Supplementary Table 4.** Blood biochemical test of nude mice after 7-day oral administration with 10⁹ CFU/day of YGMCC0564. | | | |
| --- | --- | --- | --- |
| **Parameter** | **Unit** | **Con (Mean±SD)** | **YGMCC0564 (Mean±SD)** |
| Glu-G | mmol/L | 4.47±0.83 | 4.81±0.64 |
| UREA | mmol/L | 9.59±1.25 | 9.62±1.74 |
| TG | mmol/L | 2.04±0.36 | 1.65±0.47 |
| TC | mmol/L | 2.45±0.39 | 2.48±0.33 |
| LDL-C | mmol/L | 0.23±0.07 | 0.24±0.04 |
| HDL-C | mmol/L | 1.97±0.32 | 2.03±0.28 |
| ALT | U/L | 45.34±10.31 | 41.18±7.33 |
| AST | U/L | 175.07±38.98 | 177.21±37.14 |

Abbreviations: Glu-G, glucose; UREA, urea nitrogen; TG, triglycerides; TC, total cholesterol; LDL-C, low-density lipoprotein cholesterol; HDL-C, high-density lipoprotein cholesterol; ALT, alanine aminotransferase; AST, aspartate aminotransferase. Data are presented as mean ± standard deviation (SD).

| **Supplementary Table 5.** Blood routine examination of Balb/c mice after 1-month oral administration with 10⁹ or 10¹⁰ CFU/day of YGMCC0564. | | | | |
| --- | --- | --- | --- | --- |
| **Parameter** | **Unit** | **Control** | **Low Dose**  **(10⁹ CFU)** | **High Dose**  **(10¹⁰ CFU)** |
| WBC | 10^9^/L | 8.50±1.41 | 7.23±1.17 | 7.70±1.18 |
| Neu# | 10^9^/L | 1.61±0.41 | 1.31±0.23 | 1.20±0.17 |
| Lym# | 10^9^/L | 6.38±1.11 | 5.53±0.94 | 6.11±1.03 |
| Mon# | 10^9^/L | 0.20±0.10 | 0.15±0.06 | 0.145±0.04 |
| Eos# | 10^9^/L | 0.27±0.08 | 0.22±0.03 | 0.22±0.06 |
| Bas# | 10^9^/L | 0.05±0.05 | 0.02±0.01 | 0.03±0.01 |
| Neu% | % | 18.90±3.46 | 18.15±2.76 | 15.64±1.86 |
| Lym% | % | 74.93±3.18 | 76.47±2.77 | 79.25±1.85 |
| Mon% | % | 2.23±0.92 | 2.03±0.59 | 1.86±0.35 |
| Eos% | % | 3.37±1.42 | 3.07±0.52 | 2.84±0.76 |
| Bas% | % | 0.57±0.52 | 0.28±0.11 | 0.41±0.14 |
| RBC | 10^12^/L | 10.83±0.58 | 11.17±0.22 | 10.55±1.18 |
| HGB | g/L | 183.30±9.02 | 188.40±3.50 | 173.80±20.53 |
| HCT | % | 58.71±2.83 | 60.47±1.22 | 56.99±6.15 |
| MCV | fL | 54.24±0.73 | 54.10±0.60 | 54.05±1.10 |
| MCH | pg | 16.91±0.39 | 16.88±0.18 | 16.49±0.34 |
| MCHC | g/L | 311.90±5.97 | 312.00±3.40 | 305.00±6.67 |
| RDW-CV | % | 14.69±0.22 | 14.78±0.42 | 14.84±0.51 |
| RDW-SD | fL | 31.54±0.63 | 31.74±1.19 | 31.89±1.56 |
| PLT | 10^9^/L | 819.50±82.58 | 936.20±74.81 | 1018.30±158.32 |
| MPV | fL | 6.41±0.15 | 6.64±0.27 | 6.50±0.16 |
| PDW | - | 16.01±0.17 | 15.99±0.10 | 15.85±0.18 |
| PCT | % | 0.53±0.06 | 0.62±0.04 | 0.66±0.10 |

For abbreviations, see the legend of Supplementary Table 3.*Data are presented as mean ± standard deviation (SD).

| **Supplementary Table 6.** Blood biochemical test of Balb/c mice after 1-month oral administration with 10⁹ or 10¹⁰ CFU/day of YGMCC0564. | | | | |
| --- | --- | --- | --- | --- |
| **Parameter** | **Unit** | **Control** | **Low Dose**  **(10⁹ CFU)** | **High Dose**  **(10¹⁰ CFU)** |
| Glu-G | mmol/L | 4.73±0.98 | 4.61±0.81 | 4.78±1.05 |
| UREA | mmol/L | 6.29±0.65 | 7.21±0.69 | 6.75±0.73 |
| TG | mmol/L | 1.16±0.15 | 1.02±0.18 | 1.24±0.19 |
| TC | mmol/L | 2.57±0.16 | 2.60±0.36 | 2.71±0.17 |
| LDL-C | mmol/L | 0.25±0.03 | 0.23±0.04 | 0.25±0.06 |
| HDL-C | mmol/L | 2.15±0.14 | 2.21±0.31 | 2.32±0.13 |
| ALT | U/L | 33.69±3.38 | 35.24±2.15 | 33.03±3.74 |
| AST | U/L | 157.20±25.56 | 179.43±27.41 | 158.78±22.64 |

 For abbreviations, see the legend of Supplementary Table 4.Data are presented as mean ± standard deviation (SD).

| **Supplementary Table 7.** Grouping and experimental design of the acute and chronic toxicity studies evaluating YGMCC0564 in mice | | | | | | |
| --- | --- | --- | --- | --- | --- | --- |
| Safety experiment | Group (n per group) | Species | Immune status | Sex (F/M)and number | Intervention (oral gavage, daily) | Duration |
| Acute toxicity | Con (n=20) | BALB/c nude mice | Nude (immunodeficient) | 10 F, 10 M | 0.1 mL normal saline | 3 days dosing, then 7-day observation |
| Acute toxicity | YGMCC0564 (n=20) | BALB/c nude mice | Nude (immunodeficient) | 10 F, 10 M | 1×10^9^ CFU in 0.1 mL normal saline | 3 days dosing, then 7-day observation |
| Chronic toxicity | Con (n=20) | BALB/c mice | Normal (immunocompetent) | 10 F, 10 M | 0.1 mL normal saline | one month |
| Chronic toxicity | YGMCC0564-L (n=20) | BALB/c mice | Normal (immunocompetent) | 10 F, 10 M | 1×10^9^ CFU in 0.1 mL normal saline | one month |
| Chronic toxicity | YGMCC0564-H (n=20) | BALB/c mice | Normal (immunocompetent) | 10 F, 10 M | 1×10^10^ CFU in 0.1 mL normal saline | one month |
| All mice were 6–8 weeks old and supplied by Beijing Vital River Laboratory Animal Technology Co., Ltd. Con denotes the control group receiving normal saline. YGMCC0564-L denotes the low-dose group. YGMCC0564-H denotes the high-dose group. Total animals were 40 for the acute test and 60 for the chronic test. | | | | | | |
